# A Comprehensive Study of the Genus *Sanguisorba* (Rosaceae) Based on the Floral Micromorphology, Palynology, and Plastome Analysis

**DOI:** 10.3390/genes12111764

**Published:** 2021-11-05

**Authors:** Inkyu Park, Junho Song, Sungyu Yang, Goya Choi, Byeongcheol Moon

**Affiliations:** Herbal Medicine Resources Research Center, Korea Institute of Oriental Medicine, Naju 58245, Korea; pik6885@kiom.re.kr (I.P.); songjh@kiom.re.kr (J.S.); sgyang81@kiom.re.kr (S.Y.); serparas@kiom.re.kr (G.C.)

**Keywords:** *Sanguisorba*, micromorphology, pollen, plastid genome, DNA barcode, phylogenic analysis

## Abstract

*Sanguisorba*, commonly known as burnet, is a genus in the family Rosaceae native to the temperate regions of the Northern hemisphere. Five of its thirty species are distributed in Korea: *Sanguisorba officinalis*, *S. stipulata*, *S. hakusanensis*, *S. longifolia*, and *S. tenuifolia*. *S. officinalis* has been designated as a medicinal remedy in the Chinese and Korean Herbal Pharmacopeias. Despite being a valuable medicinal resource, the morphological and genomic information, as well as the genetic characteristics of *Sanguisorba*, are still elusive. Therefore, we carried out the first comprehensive study on the floral micromorphology, palynology, and complete chloroplast (cp) genome of the *Sanguisorba* species. The outer sepal waxes and hypanthium characters showed diagnostic value, despite a similar floral micromorphology across different species. All the studied *Sanguisorba* pollen were small to medium, oblate to prolate-spheroidal, and their exine ornamentation was microechinate. The orbicules, which are possibly synapomorphic, were consistently absent in this genus. Additionally, the cp genomes of *S. officinalis*, *S. stipulata*, and *S. hakusanensis* have been completely sequenced. The comparative analysis of the reported *Sanguisorba* cp genomes revealed local divergence regions. The nucleotide diversity of *trnH-psbA* and *rps2-rpoC2*, referred to as hotspot regions, revealed the highest pi values in six *Sanguisorba*. The *ndhG* indicated positive selection pressures as a species-specific variation in *S. filiformis*. The *S. stipulata* and *S. tenuifolia* species had *psbK* genes at the selected pressures. We developed new DNA barcodes that distinguish the typical *S. officinalis* and *S. officinalis* var. *longifolia*, important herbal medicinal plants, from other similar *Sanguisorba* species with species-specific distinctive markers. The phylogenetic trees showed the positions of the reported *Sanguisorba* species; *S. officinalis*, *S. tenuifolia*, and *S. stipulata* showed the nearest genetic distance. The results of our comprehensive study on micromorphology, pollen chemistry, cp genome analysis, and the development of species identification markers can provide valuable information for future studies on *S. officinalis*, including those highlighting it as an important medicinal resource.

## 1. Introduction

The genus *Sanguisorba* L. (subtribe Sanguisorbinae, tribe Sanguisorbeae, subfamily Rosoideae, family Rosaceae), commonly known as burnet, consists of 10–33 species that are widely distributed in the Northern hemisphere [1,2,3,4,5]. Members of this genus are perennial herbs, with odd-pinnate, stipulate leaves, and densely capitate or spicate inflorescences with tetramerous, apetalous, and penicillate stigmatic flowers and dry achene fruits [4,5,6,7,8].

In East Asia, seven species of *Sanguisorba* have been described in China [4] and Japan [7], and six species in Korea [8]. Three species, namely *Sanguisorba officinalis* L., *S. stipulata* Raf., and *S. tenuifolia* Fisch. ex Link, are extensively distributed across these three countries. Moreover, the dried roots of *S. officinalis* and *S. officinalis* var. *longifolia* (Bertol.) T.T. Yu and C.L. Li are used together in herbal medicine, known as the Sanguisorbae Radix, and have been designated in the Chinese and Korean herbal pharmacopeias. In addition, *S. officinalis* is also well-known for its medicinal properties in Europe. [9,10,11,12]. Moreover, *S. officinalis* is traditionally known for its anti-allergic, anti-asthmatic, anti-inflammatory, antioxidant, antibacterial, anticancer, antiviral, and hemostatic effects [12,13,14,15,16]. However, identifying *Sanguisorba* species is challenging because of the extra-morphological similarity and sympatric distribution of the authentic medicinal resource, *S. officinalis*, and its close relatives. Thus, it is necessary to conduct comprehensive studies and integrative taxonomy to gain deeper knowledge about their biological properties.

The most recent morphological studies on *Sanguisorba* have primarily focused on selected characteristics and their evolution, including pollen [17,18] and floral morphogenesis [19]. However, comprehensive and integrative species-based analyses of the micromorphological, palynological, and molecular characteristics have not been performed to date.

The floral micromorphological characteristics observed using scanning electron microscopy (SEM) are useful taxonomic markers at various taxonomic levels and groups. They include the sepal surface of *Lathyrus* (Fabaceae) [20], the petal surface of the tribe Sorbarieae [21] and Spiraeeae (Rosaceae) [22], and the gynoecium of the genus *Arnebia* (Boraginaceae) [23]. The comparative floral morphology of the three *Sanguisorba* taxa has been studied [19]. However, the study focused on floral morphogenesis and did not include *S. stipulata* or the characteristics of the sepal cuticular surfaces.

Palynological characteristics have also been proven to be useful for systematic implications [17,18,24,25,26,27] and taxonomic delimitation [27,28,29,30] of various groups in Rosaceae. In particular, the orbicules, the small sporopollenin particles on the inner locule walls, have proven to be a taxonomically and systematically useful palynological characteristic within Rosaceae [31,32,33]. Although pollen morphology of *Sanguisorba* has been studied by Chung et al. [17] and Lee et al. [18], the critical-point drying (CPD) method, which negates the side effects of the acetolysis method, such as distortion of shape, has not been investigated. Moreover, the orbicules of *Sanguisorba* have not yet been studied.

The chloroplast (cp) is one of the most important organs in plants and functions as an essential organelle for photosynthesis, starch and fatty acid biosynthesis, and carbon fixation [34,35,36]. Genome analysis using cp, which is quick and easy to obtain, is inexpensive; it is an essential strategy in plant studies, particularly in taxonomy, plant identification, and evolutionary studies [34,35,36]. In general, angiosperms have 110–130 genes, with up to 80 protein-coding genes, 30 transfer RNA (tRNA), and 4 ribosomal RNA (rRNA) genes [34]. The cp genome has a highly conserved structure and gene content, as well as low variance compared to the nuclear and mitochondrial genomes. However, changes in the cp genome size lead to gains and losses in genes, IR expansion and contraction, and structural rearrangements [34,35,36]. The cp genomes provide useful information through comparative analysis at the family and species levels or designated comparison groups to characterize gene rearrangements, including expansion or contraction of IR regions, gene content, and gene duplication or loss. Furthermore, it enables the analysis and utilization of genetic variations due to insertions/deletions (indels), single sequence repeats (SSRs), and single nucleotide polymorphisms (SNPs). These genetic mutations can be used for species identification, classification, and evolutionary studies [37,38,39]. The remarkable reproducibility of methods based on these genetic mutation-based markers facilitated the authentication and quality control of herbal medicines [39,40,41]. Within the Sanguisorbeae tribe, several cp genomes have been reported for *S. tenuifolia*, *S. officinalis*, *S. sitchensis*, *S. filiformis*, and *Bencomia exstipulata* [42,43]. Despite being a valuable plant, there is very little genomic information available. Therefore, it is necessary to study the genome of *Sanguisorba*.

In the present study, four species of *Sanguisorba*, namely *S. hakusanensis*, *S. officinalis*, *S. stipulata*, and *S. tenuifolia*, were subjected to micromorphological and palynological analyses, and cp genomes of three species, *S. hakusanensis*, *S. officinalis*, and *S. stipulata*, were sequenced for comprehensive studies. This study aimed to (1) document and illustrate the floral micromorphology and palynology of the representative taxa using microscopy techniques; (2) characterize *Sanguisorba* cp genomes and identify genetically variable regions by comparing their global structures, (3) develop novel DNA barcodes for authenticating the species used in herbal medicines, and (4) understand the evolutionary relationships within the tribe Sanguisorbeae.

## 2. Results

### 2.1. Floral Micromorphological Characteristics of Sanguisorba

**Figure 1:**
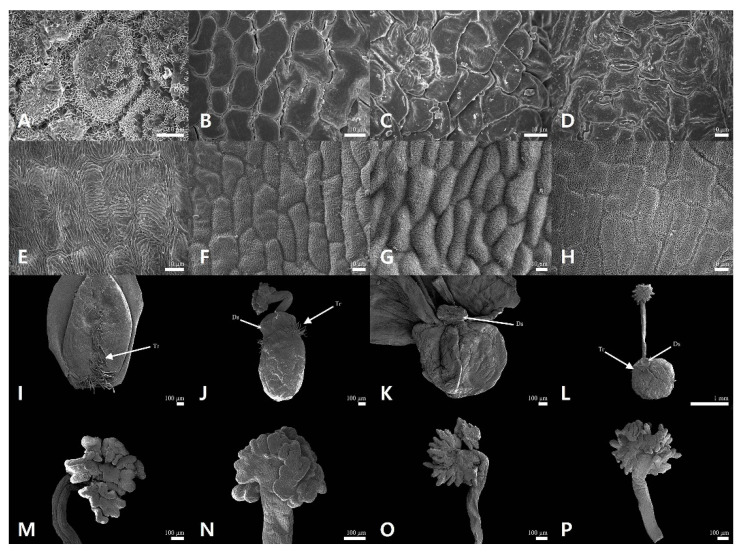
The sepal and gynoecium micromorphological characteristics of *Sanguisorba* species. (**A**,**E**,**I**,**M**) *S. hakusanensis*, (**B**,**F**,**J**,**N**) *S. officinalis*, (**C**,**G**,**K**,**O**) *S. stipulata*, (**D**,**H**,**L**,**P**) *S. tenuifolia*, (**A**–**D**) Outer sepal, (**E**–**H**) Inner sepal, (**I**–**L**) Hypanthium and gynoecium, (**M**–**P**) Stigma. DS, Disk-like structure; Tr, Trichome.

**Figure 2:**
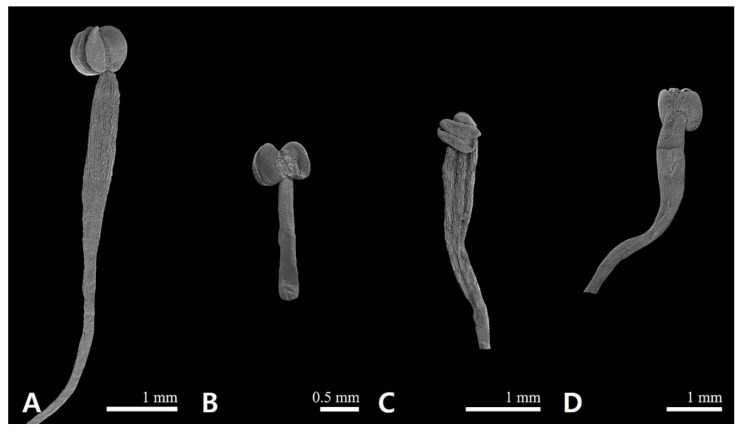
The androecium micromorphological characteristic of *Sanguisorba* species. (**A**) *S. hakusanensis*, (**B**) *S. officinalis*, (**C**) *S. stipulata*, and (**D**) *S. tenuifolia*.

The floral epidermal micromorphological characteristics (sepal, gynoecium, and androecium) are summarized in Table 1, and representative micrographs are shown in Figure 1 and Figure 2. The outer sepals of all the studied *Sanguisorba* species were smooth (Figure 1A–D), whereas those of only *Sanguisorba hakusanensis* were densely covered with platelet epicuticular waxes (Figure 1A). The inner sepal epidermis of all the studied species was similar; its cells were regularly arranged with straight to curved anticlinal walls and slightly convex with striated periclinal cell walls (cuticular folds) (Figure 2). The gynoecium was unicarpellate with a disk-like structure and comprised a unilocular ovary surrounded by a perigynous hypanthium. The shape of the hypanthium was ellipsoid in *S. hakusanensis* (Figure 1I) and *S. officinalis* (Figure 1J), and globose in *S. stipulata* (Figure 1K) and *S. tenuifolia* (Figure 1L). The disk-like structure, located on top of the hypanthium, was reduced in thickness. On the surface of the hypanthium, the non-glandular unicellular trichomes were sparse to moderate on the basal and ventral parts of *S. hakusanensis* (Figure 1I) and the upper part of *S. officinalis* (Figure 1J) and *S. tenuifolia* (Figure 1L), while they were glabrous on the surface of *S. stipulata* (Figure 1K). All the studied *Sanguisorba* species had fimbriate (penicillate, brush-like) stigma; however, the apex of the fimbriae was blunt in *S. hakusanensis* (Figure 1M) and *S. officinalis* (Figure 1N), and acute in *S. stipulata* (Figure 1O) and *S. tenuifolia* (Figure 1P). The style and stigma cell surface patterns were observed in all the studied species. *S. hakusanensis* had the longest filament (6–10 mm) with compressed dilation in the upper part (Figure 2A), whereas *S. officinalis* had the shortest (1–3 mm) with a filiform shape (Figure 2B). *S. stipulata* and *S. tenuifolia* had moderate filament lengths (5–8 mm) with compressed dilation in the upper part (Figure 2C,D).

### 2.2. Palynological Characteristics of Sanguisorba

**Figure 3:**
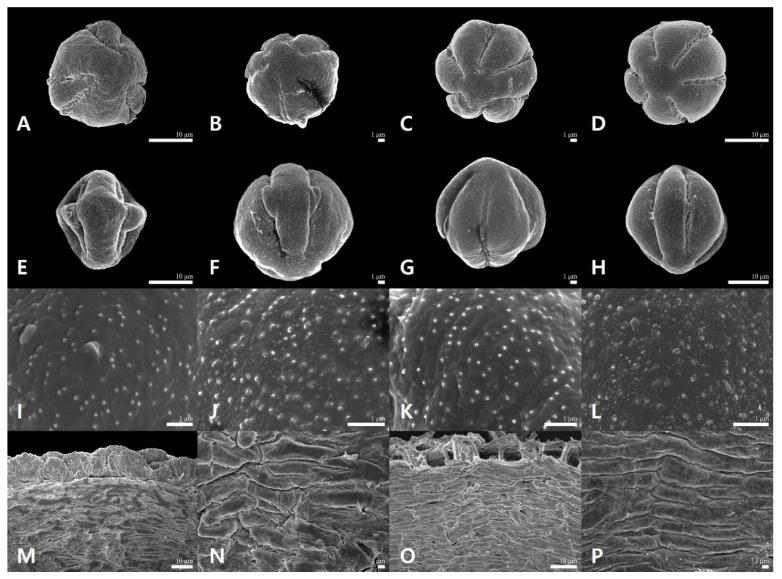
The palynological characteristic of *Sanguisorba* species. (**A**,**E**,**I**,**M**) *S. hakusanensis*, (**B**,**F**,**J**,**N**) *S. officinalis*, (**C**,**G**,**K**,**O**) *S. stipulata*, (**D**,**H**,**L**,**P**) *S. tenuifolia*, (**A**–**D**) Polar view, (**E**–**H**) Equatorial view, (**I**–**L**) Exine ornamentation, (**M**–**P**) Inner locule wall.

**Figure 4:**
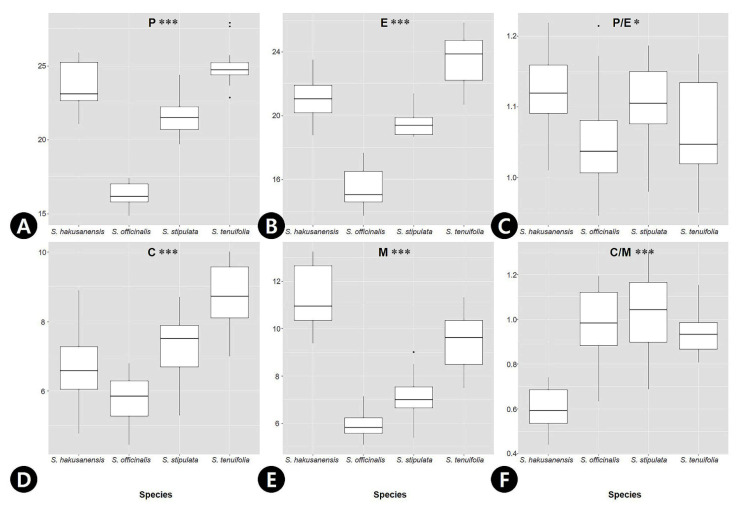
The quantitative data of the pollen morphological characteristics in *Sanguisorba* species. Boxplots show the median, 25th and 75th percentiles (box), 10th and 90th percentiles (whiskers), and outliers (closed circle). (**A**) Polar axis (P, μm), (**B**) Equatorial diameter (E, μm), (**C**) P/E, (**D**) Colpus width (C, μm), (**E**) Mesocolpus width (M, μm), (**F**) C/M. The difference of characteristics is marked as significant with an asterisk based on an analysis of variance (ANOVA) test (*; *p* < 0.05; ***; *p* < 0.001).

The palynological characteristics (pollen and orbicule) are summarized in Table 1, and representative micrographs are shown in Figure 3. The pollen grains were monads, and their size varied from small to medium (P = 14.9–27.9 μm, E = 13.8–25.8 μm; Table 1). *S. tenuifolia* had the largest pollen grains (P = 25.0 ± 1.28 μm, E = 23.5 ± 1.56 μm), while *S. officinalis* (P = 16.3 ± 0.85 μm, E = 15.5 ± 1.15 μm) had the smallest pollen grains (Table 1). The outline of the pollen grains was mostly circular in polar view (Figure 3A–D). In the equatorial view, pollen grain shapes ranged from oblate-spheroidal to prolate-spheroidal (P/E = 0.95–1.22; Figure 3E–H). Only *S. hakusanensis* had tricolporate pollen grains (Figure 3A), whereas other species had hexacolporate apertures (Figure 3B–D). The mean value of the colpus ranged from 5.85 μm (*S. officinalis*) to 8.72 μm (*S. tenuifolia*), and that of mesocolpus width ranged from 6.02 μm (*S. officinalis*) to 11.3 μm (*S. hakusanensis*) (Table 1). Three species of C/M ratio were 0.9–1.0, indicating that the widths of both the margins of each colpus and mesocolpus were nearly equal. However, the C/M ratio of *S. hakusanensis* was 0.6, indicating a wider mesocolpus than the colpus. A microechinate exine ornamentation was observed in all the studied species (Figure 3I–L). The orbicules were absent in all the species studied (Figure 3M–P). The P and E values of *S. officinalis* were lower, and their 25th and 75th percentiles did not overlap with those of the other three species (Figure 4A,B), whereas those of P/E overlapped (Figure 4C). The C value of *S. tenuifolia* was higher than that of the other species (Figure 3D). The 25th and 75th percentiles of the M and C/M values of *S. hakusanensis* did not overlap with those of the other three species (Figure 4E,F).

Based on the floral epidermal micromorphological and pollen morphological characteristics of the four *Sanguisorba* species, we provide the following identification key.
1.Outer sepal waxes platelets; hypanthium trichomes basal and ventral part; tricolporate pollen grains; mesocolpus wider than colpus*S. hakusanensis*1′.Outer sepal waxes absent; hypanthium trichomes upper part or absent; hexacolporate pollen grains; mesocolpus and colpus almost equal22.Filament length 1–3 mm and filiform shaped; hypanthium ellipsoid; stigma fimbriae blunted apex*S. officinalis*2′.Filament length 5–8 mm and compressed-dilated; hypanthium globose; stigma fimbriae acute apex33.Hypanthium trichomes absent*S. stipulata*3′.Hypanthium trichomes upper part*S. tenuifolia*

### 2.3. The Chloroplast Genome Organization of Sanguisorba

**Figure 5:**
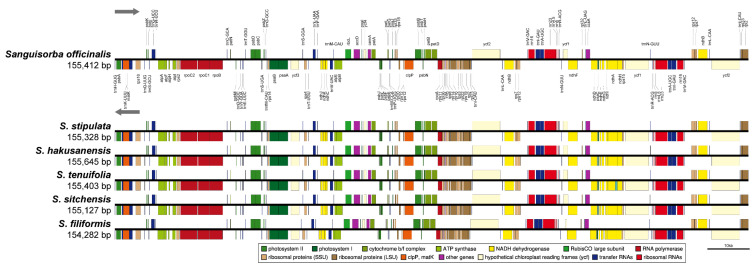
The linear gene map of the chloroplast genomes from the six *Sanguisorba* species. Genes are transcribed left to right. Genes above the line are positioned in the forward direction (left to right). Genes below the line are positioned in the reverse direction (left to right).

We sequenced three *Sanguisorba* accessions: *S. officinalis*, *S. stipulata*, and *S. hakusanensis* at approximately 380×, 805×, and 397× coverage, respectively, generating 1.5–2.6 Gb raw paired-end reads and 0.9–1.7 Gb trimmed reads (Tables S1 and S2). The complete circular cp genome was 155,328–155,645 bp and matched the quadripartite structure of cp genomes, consisting of a pair of IRs (25,562–25,593 bp) separated by the LSC (85,444–85,697 bp) and SSC (18,760–18,788 bp) regions (Figure 5, Table 2). The complete cp genomes were of high quality (Figure S1 and Table S3). The overall GC content was approximately 37%, which was slightly higher in the IR (42%) than in the LSC (35%) and SSC (31%) regions. The four cp genomes comprised 112 genes, namely 78 protein-coding, four rRNA, and 30 tRNA genes (Table 2 and Figure S2). They had 18 intron-containing genes, 15 of which had a single intron and two of which (ycf3, clpP) had three introns with duplicate genes (ndhB, trnI-GAU, trnA-UGC) in the IR regions (Tables S4–S6). Analysis of codon usage and anticodon recognition patterns revealed 26,540–26,929 codons, among which leucine, isoleucine, and serine were the most abundant (Figure S3). To identify the codon patterns, we analyzed the codon distribution in the six cp genomes using the hierarchical clustering method (Figure S4); green and red colors indicate weak (relative synonymous codon usage, RSCU > 1) and strong (RSCU < 1) codon biases, respectively. Codons with A or T at the third position had a strong codon bias. Most *Sanguisorba* displayed a similar pattern, with high RSCU values for AGA (arginine), GCT (alanine), and TTA (leucine).

### 2.4. The Repeat Sequences of the *Sanguisorba* Chloroplast Genomes

**Figure 6:**
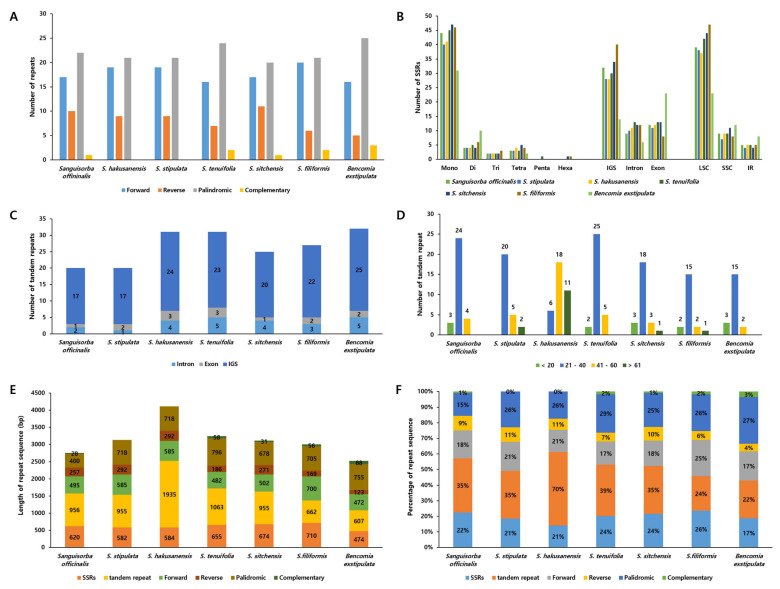
The distribution of the repeat sequences in *Sanguisorba* chloroplast genomes. (**A**) The number of single sequence repeats (SSRs) in genomic regions. (**B**) Distribution of SSRs in intergenic spacer (IGS), exon, and intron regions. (**C**) Distribution of SSR types. (**D**) Distribution of tandem repeats in genomic regions. (**E**) Distribution of tandem repeats in IGS, exon, and intron regions. (**F**) Distribution of lengths of the tandem repeats.

To determine the character and proportion of repeat sequences in the *Sanguisorba* cp genomes, we surveyed the repeat sequences of Sanguisorba, such as forward, reverse, palindromic, and complementary repeats, yielding 49–50 repeats (Figure 6A). Furthermore, we compared the differences between the genera *Bencomia* and Sanguisorba. *Sanguisorba* had a similar number of repeats. For SSRs, the mononucleotide motifs were the most abundant in all *Sanguisorba* species, followed by dinucleotide motif repeats (Figure 6B). We identified 49–60 SSRs, mostly in the LSC, particularly within the intergenic spacer (IGS) region. Most tandem repeats were located in the IGS region (Figure 6C). We found 15–25 tandem repeats that were generally 21–40 bp long in *Sanguisorba* (Figure 6D). However, unlike other Sanguisorba, *S. hakusanensis* showed longer tandem repeats. Among the tandem repeats in IGS, repeats of five regions (trnK-rps16, atpF-atpH, trnC-petN, trnT-trnL, and accD-psaI) were common in the six *Sanguisorba* cp genomes. In the total repeat sequences, although the tandem repeats were fewer than other repeat sequences, it showed the highest length, and this pattern was similar in both *Bencomia* and *Sanguisorba* (Figure 6E). The number of repeat sequences was highest for *S. hakusanensis*, with a higher number of tandem repeats (Figure 6F).

### 2.5. Comprehensive Comparative Analysis of the *Sanguisorba* Chloroplast Genomes

**Figure 7:**
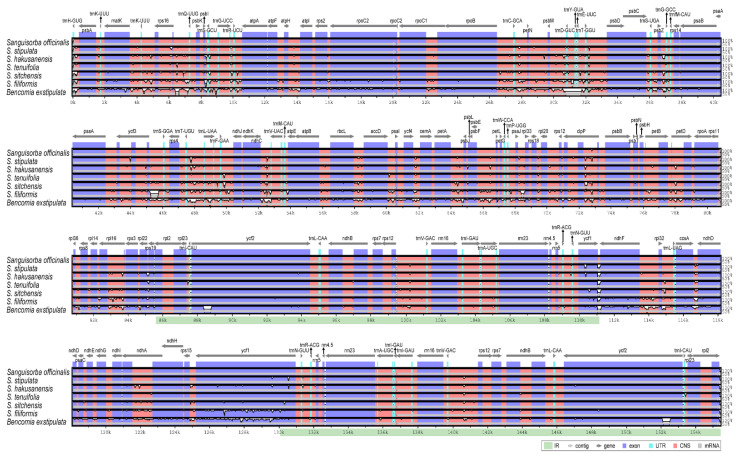
Comparison of the *Sanguisorba* chloroplast genomes using mVISTA. The complete cp genomes of six *Sanguisorba* species and *Bencomia exstipulata* were compared, with *S. officinalis* as a reference. Blue block: conserved genes, sky-blue block: transfer RNA (tRNA) and ribosomal RNA (rRNA), red block: conserved non-coding sequences (CNS). White represents the regions with sequence variation among the six *Sanguisorba* and *Bencomia exstipulata*.

**Figure 8:**
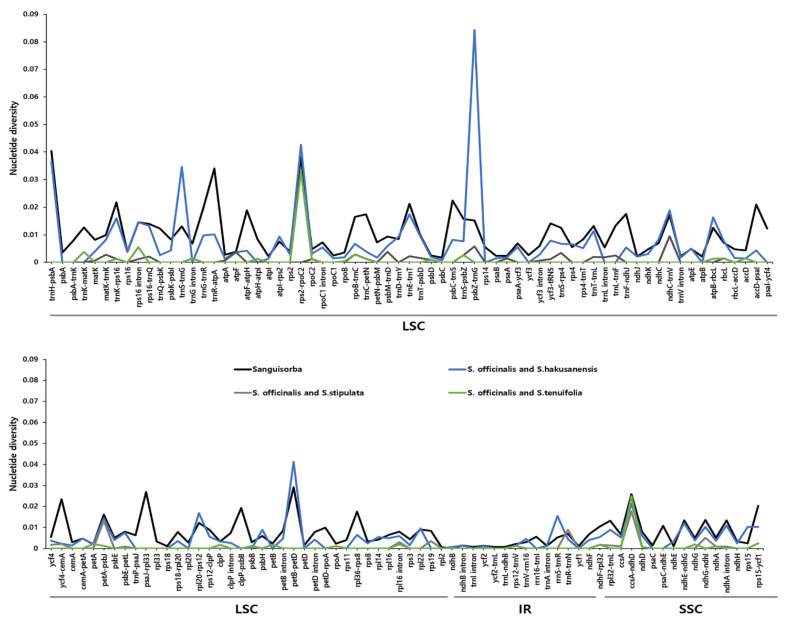
Comparison of the nucleotide diversity (Pi) values among the *Sanguisorba*. Pi value of Six *Sanguisorba* is indicated using a black line.

The sequence identities were analyzed using the mVISTA program, with the *S. officinalis* cp genome serving as a reference. Overall, the *Sanguisorba* cp genome structure was well conserved with that of *Bencomia exstipulata* (Figure 7). As expected, the genic regions were more conserved than the IGS regions when comparing each of the six species. The most divergent regions were detected in the IGS. The highest divergences were observed in the LSC and SSC regions. We analyzed genetic divergence using six *Sanguisorba* cp genomes, and species-specific mutations were detected using pairwise alignment by criterion in *S. officinalis* (Figure 8). We determined the Pi values for the six *Sanguisorba* cp genomes and species-specific Pi values. Overall, the *Sanguisorba* cp genome had an average Pi value of 0.008. The *Sanguisorba* cp genomes had divergent regions in the non-coding *trnH-psbA*, *trnR-atpA*, *rps2-rpoC2*, and *petB-petD* in LSC and *ccsA-ndhD* in SSC. Furthermore, the *trnH-psbA* had the highest Pi value of approximately 0.04, followed by *trnR-atpA* (0.034) in the LSC regions. The species-specific mutations in *S. officinalis* and *S. hakusanensis* had the highest Pi values in *psbZ-trnG* (0.082) and *petB-petD* (0.041). However, the Pi values of *S. officinalis* and *S. stipulata* were more conserved than those of the other *Sanguisorba* species. As expected, the IR regions were more highly conserved than the single-copy regions. We also analyzed the syntenic regions and sequence identities within the six *Sanguisorba* species and *Bencomia exstipulata* (Figure S5). A few genome size variations were observed in *S. filiformis*. Overall, we found that the cp genomes of *Sanguisorba* were highly conserved in the genome structure and gene order compared to that of *Bencomia exstipulata*.

We compared the IR contraction and expansion in the *Sanguisorba* cp genome (Figure S6). Similar IR lengths (ranging from 25,562 to 25,615 bp) and differences in the IR expansions and contractions were observed. The rps19 gene was observed to be located entirely in the LSC region. The ycf1 gene spanned the IRb/SSC and SSC/IRa junctions. The IRb/SSC border was also located in the ycf1 gene, although it extended by the same length into the *ycf1* gene. Overall, the IR was found to be expanded in all the cp genomes assessed. The *trnH* gene was located in the LSC region, 2–7 bp from the IRa/LSC boundary. The *rpl2* genes were duplicated in the IR regions flanking the border junctions. Generally, the *Sanguisorba* cp genome was highly conserved.

**Figure 9:**
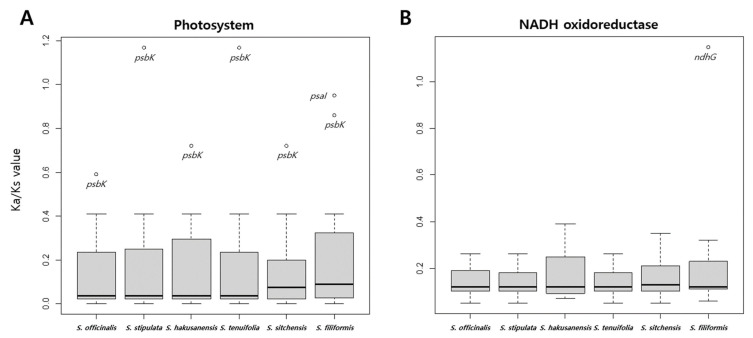
Ka/Ks values for (**A**) the photosystem- and (**B**) NADH oxidoreductase-related genes in the *Sanguisorba* cp genomes.

Based on the non-synonymous substitution to synonymous substitution (Ka/Ks) ratio, 78 genes were identified that showed evidence of selective pressure in the *Sanguisorba* cp genomes (Figure S7). Most genes were conserved and exhibited relaxed selection (0 < Ka/Ks ratio < 1). No significant gene evolution was observed according to the regional grouping (i.e., the LSC, IR, or SSC regions). Within the *Sanguisorba* cp genomes, 53 genes had Ka and Ks values of >0.001. The average Ka and Ks values were 0.0087 and 0.0546, respectively. The highest Ks value for *rpl2* across the *S. sitchensis* cp genome was 0.1796. The highest Ka/Ks ratios for *psbK* in *S. stipulata* and *S. tenuifolia* were 1.168 (Figure 9A). Among the *Sanguisorba* species, the *psbK* genes of *S. stipulata* and *S. tenuifolia* showed evidence of positive selection. In addition, *S. filiformis* showed positive selection for *ndhG* (Ka/Ks ratio of 1.148) (Figure 9B). Thus, although the organization of the *Sanguisorba* cp genomes was highly conserved, positive selection pressures (Ka/Ks > 1) were observed for *psbK*. Unlike other gene groups, *psbK* of the six *Sanguisorba* cp genomes showed varied patterns (Figure 9 and Figure S8).

### 2.6. New DNA Barcode for the Authentication of Sanguisorbae Radix

The dried roots of *S. officinalis* and *S. officinalis* var. *longifolia*, collectively called Sanguisorbae Radix, are used as traditional herbal medicine in Korea [10]. *S. officinalis* and *S. officinalis* var. *longifolia* has been frequently mistaken with other similar *Sanguisorba* species due to similar morphological characterization, particularly as dried roots or powdered products. Therefore, SR should be distinguished to ensure the quality and efficacy of herbal medicines. We performed DNA barcoding analysis for distinguishing four species and one variety using the Internal transcribed spacer (ITS), *matK* (marker name; S_matK), *rbcL* (S_rbcL), *ycf1* (Y1-1, Y1-2), *matK-rps16* (MR16), and *ycf3* intronic (Y3) regions (Figure S9). We obtained the sequences of four species and one variety consisting of ten samples (Table S7). In ITS, we did not find any nucleotide sequences to distinguish the *Sanguisorba* species (Figure S10). A total of six regions were considered for identifying the species-specific divergent regions. As *Sanguisorba* is not amplified with the universal DNA barcode primers, *matK* and *rbcL*, we designed and applied primers from the cp genome information (Figure S8). S_matK, S_rbcL, MR16, and Y1-1 had the most variable sites (P = 0.00296 for S_matK, 0.002 for S_rbcL, 0.00466 for MR16, and 0.00699 for Y1-1) among the four species and one variety (Table 3). They exhibited 5 (1.2% in MR16) to 7 (0.8% in S_rbcL) parsimony informative sites. The number of haplotypes was indicated to be one to four. Thus, there were no mutations that could cover the four species and one variety in one region. The three regions (*matK-rps16*, *ycf1*, and *ycf3* intron) could represent the species-specific variability using indel mutations (Table 3 and Figure S11). The aligned DNA barcode sequences in the six *Sanguisorba* are shown in Figure S11. *S. stipulata* could be identified with a unique nucleotide in the S_matK region of 689 bp. The S_rbcL marker yielded 870 bp region, distinguishing four nucleotides from *S. hakusanensis* and one nucleotide from *S. officinalis*, and thereby could be used to discriminate *S. hakusanensis* and *S. officinalis* from other *Sanguisorba* species. Indel variants of *S. hakusanensis* were distinguished using the MR16 marker. Y1-1 yielded a 214 bp region, with one nucleotide for distinguishing *S. officinalis* var. *longifolia*, which was distinct from that of *S. hakusanensis* and *S. stipulata* for Y3.

### 2.7. The Phylogenetic Relationships among Sanguisorba

**Figure 10:**
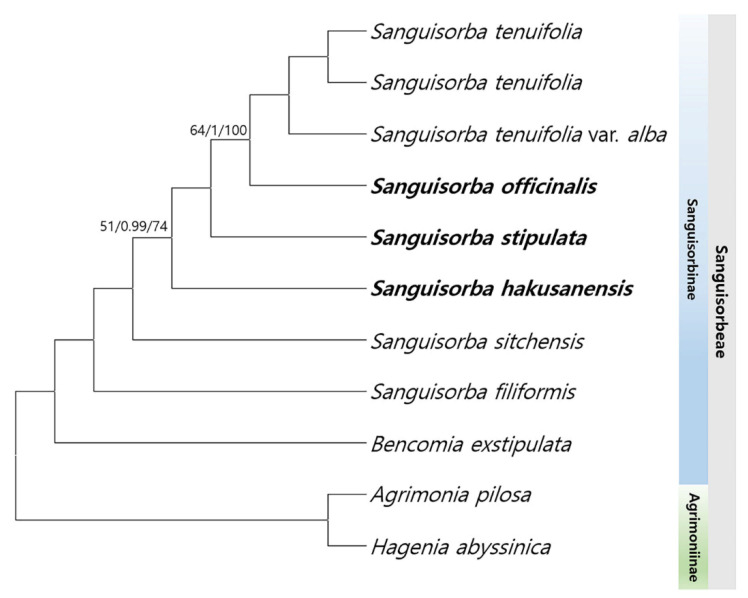
Phylogenetic tree from the *Sanguisorba* using maximum parsimony (MP), Bayesian posterior probabilities (PP), and maximum likelihood (ML) bootstraps. Topology is shown with MP bootstrap values/Bayesian PP/ ML bootstrap support values given at each node. MP and ML values of 100% and Bayesian inference (BI) PP of 1.0 are not marked. The cp genomes completed in this study are indicated with bold text.

To verify the phylogenetic relationships among the members of the *Sanguisorba*, we identified 57 conserved protein-coding sequences by aligning 55,760 bp shared by all the six *Sanguisorba* species with *Agrimonia pilosa* and *Hagenia abyssinica* serving as outgroups (Figure 10). The maximum likelihood (ML) and Bayesian inference (BI) topologies were highly congruent with the whole genome sequences and CDS datasets (Figure S12). The phylogenetic analyses strongly supported all but one lineage (ML = 100%, BI = 1.0). Six *Sanguisorba* in the tribe clustered well. *S. tenuifolia*, *S. officinalis*, and *S. stipulata* formed a monophyletic group. *S. hakusanensis* was positioned as a sister group. Overall, the clustering revealed that *S. tenuifolia* and *S. officinalis* shared the closest phylogenetic relationship, with *S. filiformis* as their sister group and *Bencomia exstipulata* distinct from *Sanguisorba*.

## 3. Discussion

*Sanguisorba* species showed substantial diversity with respect to the androecium (filament), gynoecium (stigma), the hypanthium, and pollen grains (aperture and colpus pattern). However, all studied species shared similar cellular surface patterns of floral parts such as sepal, hypanthium, style, stigma, exine ornamentation, and orbicule distribution. The members of the *Sanguisorba* are apetalous flowers (lacking petals), and their sepals look like petals. The surface pattern of sepal, smooth on the outer (abaxial) and slightly convex with striation on the inner (adaxial) surface, was similar to that of other insect-pollinated flowers [44]. A recent study suggested a possible shift in the pollination mechanisms in this genus because both anemophilous and entomophilous characters are present [19]. Thus, our sepal micromorphological data of *Sanguisorba* may provide additional evidence of insect pollination as a role of petals.

Epicuticular waxes are hydrophobic substances present on the surfaces of land plants. Their various shapes and distribution patterns, especially on the leaves and stem surfaces, have proven useful for taxonomic studies [45,46,47,48,49,50,51]. Recently, the floral waxes of sepals, petals, and labella of the following genera have provided valuable diagnostic characteristics: *Justicia* [52], *Corybas* [53], *Crepidium* [54], and *Paphiopedilum* [55]. In this study, the platelet waxes were only found on the outer sepals of *S. hakusanensis*. We identified the possibility of taxonomical application of the floral waxes, although further studies including all the *Sanguisorba* species are required. Moreover, epicuticular waxes, as direct plant interfaces, reportedly have multifunctional properties [56]. In particular, they control the water loss and reduce transpiration rates [57], and epicuticular waxes are one of the xeromorphic cuticle features [58]. Among the studied species, floral wax is only found in the *S. hakusanensis* growing in the alpine region. Thus, further study of the ecological implications of the leaf epicuticular waxes in *Sanguisorba* is also required.

The hypanthium and stigma characteristics were helpful for the identification of the *Sanguisorba* species. Four *Sanguisorba* species could be distinguished into two groups based on the shape of the hypanthium: ellipsoid and globose. This pattern is consistent with the observations of Wang et al. [19] for *S. tenuifolia* var. *alba* (their Figure 3), *S. hakusanensis* (their Figure 7E), and *S. officinalis* (their Figure 9). However, the disk-like structures and style shapes were not consistent. The degree of development of the disk-like structure was different for *S. tenuifolia* (Figure 1L) and *S. tenuifolia* var. *alba* (Figure 4F). *S. tenuifolia* var. *alba* had a thickened disk-like structure, unlike that of *S. tenuifolia*. To confirm their taxonomic usefulness, research that covers all species is required. Although almost all samples collected at anthesis were observed in the bent style, some flowers of the same individual had a straight style. Thus, this feature was inconsistent and unusable for identification. All the *Sanguisorba* species have a fimbriate stigma [4,5,6,7,8]; however, their fimbriae lobation and apex shape differed. The stigma surface area is closely related to the pollination mechanisms and efficiency [59]. Thus, a more detailed correlation between the stigma receptivity, different shapes, and surface areas and their pollination efficiency in this genus is required.

Most androecium characters, such as dorsifixed anthers, striation surface cell pattern of the outer anther, and filament, were similarly shared among all the studied species. However, *S. officinalis* could be easily distinguished from other species based on the shortest (shorter than sepal) and filiform filaments. The remaining three species had relatively long (longer than sepal) and compressed-dilated filaments. Long filaments exposing anthers are efficient for wind pollination from an aerodynamics perspective, particle transport by air, and capture [59,60]. Thus, differences in the filament characteristics might be a result of selection by different pollinators. Verification of the relationship between the pollination mechanism and filament shape is needed to understand their adaptation and pollination biology.

The palynological variation of the studied species is less diverse, except for the aperture number. Although most of the Rosaceae taxa had tricolporate pollen [28,29,61,62], tetra- and hexacolporate pollen were also found in the tribe Sanguisorbeae [17,18]. The tri-colporate pollen of *S. hakusanensis* and hexacolporate pollen of *S. tenuifolia* and *S. stipulata* in this study are consistent with the observations of Lee et al. [18]. According to the authors, the aperture of *S. officinalis* has been recognized as tricolporate [17,27,61] or hexacolporate [63,64,65]. Thus, the aperture type of *S. officinalis* pollen grains remains controversial. Most *S. officinalis* pollen grains were observed as hexacolporate, although some were confused for tricolporate or hexacolporate. The apertures are where the pollen tube emerges and breaks through the wall at the time of germination [66,67]. Thus, *S. officinalis* pollen grains, which have places for six emerging pollen tubes, are hexacolporate from a plant growth point of view.

Orbicules (or Ubisch bodies) are small and granular particles found in the inner locule wall of mature anthers and are composed of sporopollenin [68,69,70,71]. In the Rosaceae, the occurrence of orbicules represents the consistency within the tribe in Sorbarieae [31], Neillieae [32], and genera in *Luetkea*, *Sibiraea*, and *Xerospiraea* [33]. Orbicules have been consistently absent in all the taxa investigated; therefore, the absence of orbicules in *Sanguisorba* might be a possible synapomorphic character.

In this study, we determined the complete cp genomes of three *Sanguisorba* species. The *Sanguisorba* cp genomes were similar to those of the other Sanguisorbeae cp genomes, based on a typical quadripartite structure. The *Sanguisorba* cp genomes were found to have 112 unique genes; however, their gene order, GC content, genomic structure, and overall length (155,403–155,644 bp) were within the range of the previously described Sanguisorbeae cp genomes [42,43,72,73]. Codon usage is a key factor in the correct expression of genetic information, and it plays an important role in shaping cp genome evolution [74,75]. The *Sanguisorba* have nearly the same codons as the cp genomes of *Bencomia exstipulata*. The RSCU values indicated synonymous codon usage bias, with a disproportionate number of amino codons having an A or T as their third nucleotide. This phenomenon has been observed in many angiosperm cp genomes [74,76,77]. We also surveyed the RSCU values of the *Sanguisorba* cp genomes. Half of the codons had a weak codon bias (Figure S4), denoted in green in the figure (RSCU > 1). This result is similar to that of other cp genomes, and most codons with high RSCU values had an A or T in the third position of their amino acid. In particular, the TAC (tyrosine), GAC (aspartic acid), and CGC (arginine) codons had relatively low RSCU values. The RSCU values of the *Sanguisorba* cp genomes were consistent with those of the other higher plants. The high RSCU values corresponded to the more highly conserved cp genes.

The repeat sequence affects the length of the genome and is one of the factors that indicate the characteristics of the genome, such as structural variation, gene loss, orientation, and duplication [34,78,79]. We analyzed the repeat sequence of *Sanguisorba* by dividing it into six types (forward, reverse, complementary, palindromic, SSRs, tandem repeat) compared to that of *Bencomia* (Figure 6). In the case of general repeats, *Sanguisorba* was similar to *Bencomia*, and there was very little difference between the species. General repeats appeared in a small proportion of the entire repeat sequence. In the SSR consisting of 1–6 nucleotides, mononucleotide repeats occupied the most in the single-copy regions (LSC, SSC). This finding agrees with previous reports, where the most mononucleotide repeats were A and T repeats due to an abundance of polyamines and polythymines in the cp genome [37,80]. Defining SSRs in *Sanguisorba* cp genomes could provide useful genetic resources for species identification and population genetics. Tandem repeats of 2 to 4 nucleotides long were detected in the repeat sequences of the *Sanguisorba* cp genomes, most of which were 20–40 bp in length. Tandem repeats in *S. hakusanensis* were significantly more compared to other species and accounted for 70% of the total length of the repeat. The tandem repeats of *S. hakusanensis* might indicate that the genetic variation of this species is greater than that of other *Sanguisorba* species. The tandem repeats in other *Sanguisorba* species accounted for more than 35% of the total repeats, and *S. filiformis* had similar tandem and forward repeat proportions. Overall, the tandem repeats of *Sanguisorba* showed a significant difference compared to that of *Bencomia* and showed unique characteristics of the *Sanguisorba* cp genomes.

The mVISTA results showed that the cp genomes of the *Sanguisorba* species have low diversity. Their genic regions are more conserved than their IGS regions, with the latter observation being consistent with that previously observed for cp genomes of the same genus [43]. In terms of nucleotide diversity, most divergent regions are non-coding, which is generally consistent with that for other cp genomes. Other cp genomes were reported to have highly variable non-coding sequences at *trnH-psbA*, *trnR-atpA*, *rps2-rpoC2*, and *petB-petD* in the LSC regions and at *ccsA-ndhD* in the SSC regions (Figure 8). These regions were previously found to be hotspots for genetic variation [81,82,83,84]. We performed a detailed comparative analysis for each species, using *S. officinalis* as the standard because *S. officinalis* is an important herbal medicine resource. As expected from the phylogenetic relationship, *S. officinalis* was similar to *S. tenuifolia*. They showed little difference, except in some regions. However, *S. officinalis* and *S. hakusanensis* showed the greatest differences, especially in the species-specific divergent region (*psbZ-trnG*). This area was mainly conceived as poly (A), poly (T), and poly (AT) and exhibited a large variation under the influence of this repeat. Compared to other species, it appeared to be species-specific for *S. hakusanensis*. We noticed that the repeat sequences affected the diversity. Overall, the genetic variations showed very similar patterns within the Sanguisorbeae family. Our results indicate that the hotspot region includes these variable regions. The IR regions are known to be more highly conserved than the single-copy regions. The hotspot regions of plant species in the Sanguisorbeae family indicate underlying evolution and can be used as DNA barcodes to distinguish species or genera, depending on the variabilities of the regions.

The IR contraction and expansion in the angiosperm cp genomes causes the cp genome size to vary [85]. Previous studies have identified extremely short IRs or the loss of the IR regions and genes [86,87]. The family Sanguisorbeae is highly conserved in terms of the IR length and position. However, we found that the lengths of the IR regions ranged from 25,562 to 25,609 bp, indicating that some contraction/expansion occurred in the IR regions (Figure S6). The *rps19* gene is located in the LSC region, and ycf1 was identified at the IR/SSC junction; *ndhF* was positioned in the SSC region based on the overlapping region in *Bencomia exstipulata*. Thus, the *Sanguisorba* cp genome has a contracted IR. This phenomenon has been observed in other *Sanguisorba* cp genomes [43]. *rpl2* exhibited gene duplication in IRs compared to the dicotyledon cp genomes [34]. Although the genome structure of the IR region was highly conserved among the cp sequences of the *Sanguisorba* species, extreme gene shifting and duplication have occurred in the *Sanguisorba* genus. Taken together, these findings indicate that the cp genomes of the *Sanguisorba* members are highly similar and conserved.

To determine the selection pressure in the protein-coding genes, the Ka/Ks ratios from the collinear genes were examined as evolutionary markers (Figure S7). The most conserved genes exhibited purifying selection (Ka/Ks ratios, 0–0.183). The *Sanguisorba* members had two positively selected genes, *ndhG* and *psbK*, which underwent selective pressure in the *S. filiformis*, *S. stipulata*, and *S. tenuifolia* cp genomes, respectively. There is an indication of species-specific selection pressures. The positive selection pressures for the *ndhG* and *psbK* genes are commonly observed in the angiosperm cp genomes [88,89,90,91]. The *ndhG* genes encode for one of the eleven *ndh* subunits of the Ndh1-complex. The *psb* genes encode the photosystem II that consists of 17 subunits. Among them, 15 subunits are present in the cp genome [34]. Genes showing positive selection are considered to undergo adaptive evolution in response to their environment. To confirm the species-specific positive selection, the pattern of positive selection of the gene group was confirmed (Figure 9 and Figure S8). *ndhG* was predicted to be affected by the species-specific selection pressure. However, among the photosystem II-related genes, the *psbK* gene was affected by the selection pressure compared to other genes, and it was possible to confirm that it was affected at the genus level. In particular, *S. filiformis* showed a higher level of selection pressure than *psbK*, although *psbI* was also a purifying selection factor. As a result, it was shown that *Sanguisorba* was influenced by the environment at the time of differentiation. When looking at the results of *ndhG* and *psbI* of *S. filiformis*, it is inferred that the selection pressure by the environment acted after species differentiation.

Beyond these results, *Sanguisorba* showed no dynamic variants in the cp genome compared to *Bencomia*. The cp structure and gene order were very similar, with some genes associated with a positive selection pressure. These findings provide evidence of adaptation to environmental changes during the evolutionary process of *Sanguisorba* and show differences at the tribe and the family levels. The gene-selection pressure suggests that the genes of interest are undergoing essential adaptations to their environment.

Differentiation of herbal medicines and species identification of plant origin are recognized as important factors in the standardization of herbal medicines [23,39]. As molecular markers are a very efficient and objective method compared to morphological discrimination, they are used as essential elements for the differentiation of oriental medicines or species identification. In particular, DNA barcodes are widely used to identify herbal medicines [92,93,94]. In addition, because information on the cp genome can be easily recognized with the development of NGS technology, many studies have reported the development of markers using genetic variations such as indels and SNPs through genome comparison [95,96,97].

We developed new DNA barcode sets to distinguish the authentic and adulterant Sanguisorba. The dried roots of *S. officinalis* and *S. officinalis* var. *longifolia*, used in traditional herbal medicine in Korea was studied [10,11]. These are valuable herbal medicinal plants that are indiscriminately used with other *Sanguisorba* species. They are frequently used without species identification because their root shape is similar to that of other *Sanguisorba* species. First, we analyzed the ITS regions; however, they are highly conserved, without genetic variants in *Sanguisorba*. A previous study showed that 18S-5.8S-26S rDNA sites are highly conserved in *Sanguisorba* caused by common polyploidization in Rosaceae [97]. The species-specific nucleotides did not appear in the ITS region because of the ploidy effect of *Sanguisorba*. For efficient barcode analysis, ploidy analysis of the relevant species or acquisition of prior information is required. Thus, *Sanguisorba* is not suitable for the identification of species with ITS barcodes. Therefore, we applied a universal and novel DNA barcode region in the cp genome. *matK* and *rbcL* are widely used as universal DNA barcodes. A previous study reported that *matK* and *rbcL* primers were not amplified in *Sanguisorba* [97]. Therefore, we designated a stable region without mutation as a primer based on information obtained from the cp genome, and a stable amplification product was obtained from S_matK and S_rbcL as well as other regions such as *matK-rps16* (MR16), *ycf1* (Y1-1, Y1-2), and *ycf3* intron (Y3). Our newly developed Sanguisorba-only DNA barcodes can ultimately discriminate between *S. officinalis* and *S. officinalis* var. *longifolia*. In particular, the *ycf1* gene had a unique nucleotide for *S. officinalis* and *S. officinalis* var. *longifolia*. In addition, *S. hakusanensis* had multiple barcode regions in the *matK*, *rbcL*, *matK-rps16*, and *ycf3* introns. We have successfully developed molecular markers that can distinguish *S. officinalis* and *S. officinalis* var. *longifolia* from the other *Sanguisorba* species.

The marker developed by utilizing the information obtained from the cp genome will be a valuable tool for quality check or species identification of the *Sanguisorba* species and Sanguisorbae Radix. It will be of great help in the production of highly pure herbal ingredients.

The cp genomes provide valuable genomic resources for accurately determining phylogenetic relationships, particularly among the closely related species and unresolved taxa, because of their infrageneric complexity and the differences in species concepts among authors [23,98]. Previous studies have indicated weak relationships among the *Sanguisorba* species in Rosaceae because they used insufficient genetic information. Meng et al. [43] presented a phylogenetic study of *Sanguisorba*, which included 10 *Sanguisorba* species based on the cp loci in the *matK* region. *S. hakusanensis* and *S. stipulata* as well as *S. officinalis* and *S. tenuifolia* were monophyletic. The present study found them to be paraphyletic with a high resolution. Meng et al. [43] reported phylogenetic relationships for the cp genomes of the four *Sanguisorba* species. These results are consistent with those of our study (Figure 10).

As described above, since *Sanguisorba* shows characteristics of a highly conserved cp sequence, it is very difficult to analyze the species relationship via phylogenetic analysis with some cp loci. In our results, the ML and BI trees had a lower resolution in *S. officinalis* and *S. stipulata* (Figure S10). However, our results demonstrated the species identification and phylogenetic relationship of *Sanguisorba*. This study can be the basis for research on *Sanguisorba* and the tribe Sanguisorbeae. Although additional analysis is warranted, it is a valuable study in a context where very little information about the genome of *Sanguisorba* is available.

## 4. Materials and Methods

### 4.1. Plant Materials

The plant material used in this study was collected from the natural populations. Original descriptions and other relevant taxonomic literature were searched and used for the accurate identification of all the studied species.

Identification numbers were assigned to all the samples. The voucher specimens were deposited in the Korean Herbarium of Standard Herbal Resources (Index Herbariorum code: KIOM) at the Korea Institute of Oriental Medicine, Naju, Korea. Information about the samples used for micromorphological, palynological, and plastid genome sequence analyses and marker tests are listed in Table S7.

### 4.2. Microscopic Observations

For detailed floral micromorphological and palynological observations, fully mature reproductive organs were examined using a stereomicroscope (SZX16; Olympus, Tokyo, Japan). All the studied materials were in the same anthesis stage. For SEM, the dried samples from the voucher specimens were rehydrated overnight in a wetting agent (Agepon: distilled water, 1:200) (Agfa Gevaert, Leverkusen, Germany). The floral samples, including pollen, were dehydrated using a graded series of ethanol (50, 70, 90, 95, and 100% ethanol) at room temperature (25 °C) for 1 h at each ethanol concentration. The dehydrated material was immersed in liquid CO_2_ for CPD (SPI-13200JE-AB; SPI Supplies, West Chester, PA, USA) and subsequently mounted on aluminum stubs using a double-sided adhesive conductive carbon disk (05073-BA; SPI Supplies, West Chester, PA, USA). All samples were gold-coated using an ion-sputtering device (208HR; Cressington Scientific Instruments Ltd., Watford, UK) and observed using a low-voltage field-emission scanning electron microscope (JSM-7600F; JEOL, Tokyo, Japan) at an accelerating voltage of 5–10 kV and a working distance of 5–10 mm.

The measurements were based on at least 20 pollen grains. Six variables, namely P (polar axis), E (equatorial diameter), P/E, C (colpus width), M (mesocolpus width), and C/M, were determined based on the SEM micrographs and measured using the Digimizer software (Digimizer version 5.4.3, MedCalc Software, Mariakerke, Belgium). To demonstrate the variations in these variables, species boxplots were generated using the ggplot2 library [99] in the R package (version 3.6.3; R Foundation for Statistical Computing, Vienna, Austria). Moreover, a one-way analysis of variance (ANOVA) was also performed to test the significance of differences in quantitative pollen data. The pollen terminology was based on those proposed by Punt et al. [66] and Verstraete et al. [100].

### 4.3. Genome Sequencing and Assembly

The total DNA of the four tested species was extracted using a modified CTAB method [101]. Four libraries were prepared from the total genomic DNA using the TruSeq DNA Nano Kit following the manufacturer′s protocols and the NextSeq500 platform (Illumina, San Diego, CA, USA), generating 1.5–2.6 Gb of paired-end (2 × 150 bp) reads. The generated reads were trimmed, and their quality was checked using the CLC quality trim implemented in the CLC Assembly Cell software (version 4.2.1; CLC Inc., Aarhus, Denmark). The trimmed paired-end reads (Phred score ≥ 20) were assembled using the CLC genome assembler (ver. 4.2.1; CLC Inc.) with default parameters. The SOAP de novo gap was used to fill the gaps based on the alignment of paired-end reads [102]. The contigs were aligned against the NCBI nrDB to detect cp contigs and were retrieved from the total contigs using Nucmer [103]. The aligned contigs were ordered using the cp genome sequences of *Rosa odorata* (KF753637), *S. officinalis* (MF678801), *S. filiformis* (MF678800), *S. stipulata* (MF678798), and *S. tenuifolia* var. *alba* (MF678799) as the reference. The complete cp genomes were validated by PCR-based sequencing using sequence-specific primers (Table S8). The PCR products of the four junctions (LSC/IRa, IRa/SSC, SSC/IRb, and IRb/LSC) were compared to complete the cp genome sequences. Finally, the total trimmed paired-end reads were mapped onto the complete genome sequences using BWA ver. 0.7.25 [104] (Figure S1). The new cp genome sequences of *S. officinalis*, *S. stipulata*, and *S. hakusanensis* obtained in this study were deposited in the NCBI GenBank database under the accession numbers MZ145057, MZ145058, and MZ145059.

### 4.4. Genome Annotation and Comparative Analysis

The gene annotation of the *S. officinalis*, *S. stipulata*, and *S. hakusanensis* cp genomes was performed using GeSeq [105], and the annotation results were concatenated using an in-house script pipeline. The protein-coding sequences were manually curated and confirmed using Artemis [106] and checked against the NCBI protein database. The tRNAs were confirmed using the tRNAscan-SE 1.21 [107]. The IR region sequences were confirmed using an IR finder and RepEx [108]. Circular maps of the *S. officinalis*, *S. stipulata*, and *S. hakusanensis* cp genomes were obtained using OGDRAW [109]. The GC content and RSCU of the four cp genomes were analyzed using the MEGA7 software [110]. The codon usage distribution of six *Sanguisorba* cp genomes was visualized using the Heatmapper program, with the average linkage of clustering and Euclidean distance measurement methods [111]. An RSCU < 1.00 indicated a codon that was used less frequently than expected, whereas an RSCU > 1.00 indicated a codon that was used more frequently than expected. The MAUVE V2.3.1 [112] was used to identify local collinear blocks of seven Sanguisorbinae (*S. officinalis*, *S. stipulata*, *S. hakusanensis*, *S. tenuifolia*, *S. sitchensis*, *S. filiformis*, and *Bencomia exstipulata*). The mVISTA program in the Shuffle-LAGAN mode was used to compare the cp genomes with Rosa odorata (KF753637) as a reference. The DnaSP version 6 [113] was used to calculate the nucleotide variability (Pi) among the cp genomes. To confirm the exact genetic variants, the CDS, introns, and IGS regions were analyzed separately. The substitution rates Ka and Ks of six *Sanguisorba* species were estimated using the KaKs Calculator ver. 2.0 [114] with *Bencomia exstipulata* as a reference.

### 4.5. Repeat Analysis

We used the REPuter to find forward, reverse, palindromic, and complementary repeats with a minimal length of 20 bp, an identity of 90%, and a hamming distance of 3 [115]. The SSRs were detected using MISA [116], with the minimum number of repeat parameters set to 10, 5, 4, 3, 3, and 3 for mono-, di-, tri-, tetra-, penta-, and hexanucleotides, respectively. Tandem repeats ≥20 bp were identified using the Tandem repeats finder [117] with a minimum alignment score of 50 and a maximum period size of 500; the identity of repeats was set to ≥90%.

### 4.6. Development of New DNA Barcodes for Distinguishing Sanguisorba

To detect species-specific variation, the genetic variant region was confirmed through mVISTA, nucleotide diversity, and manual direct sequence analysis. The candidate regions for distinguishing *Sanguisorba* selected indel or SNP regions. We constructed primers to determine whether the candidate region was amplified and to detect the genetic variant differences of each species. The primers flanking the five variable regions were designed using the Primer-BLAST tool (https://www.ncbi.nlm.nih.gov/tools/primer-blast/ accessed on 15 June 2021). The specificity of these markers was confirmed using PCR amplification with 20 ng of genomic DNA extracted from the samples of five *Sanguisorba* species and one variety (*S. officinalis* and *S. officinalis* var. *longifolia*, *S. hakusanensis*, *S. stipulata*, and *S. tenuifolia*) in a 20 μL PCR mixture (Solg™ 2× Taq PCR smart mix 1, Solgent, Daejeon, Korea) with 10 pmol of each of the *matK* (primer name: S_matK), *rbcL* (S_rbcL), *matK-rps16* (MR16), *ycf1* (Y1-1, Y1-2), and *ycf3* intron (Y3) primers. The reaction was carried out on a ProFlex PCR system (Applied Biosystems, Waltham, MA, USA) with the following amplification parameters: initial denaturation at 95 °C for 2 min; 35 cycles at 95 °C for 40 s, 58–62 °C for 40 s, 72 °C for 50 s, and a final extension at 72 °C for 5 min. The PCR products were separated on a 2% agarose gel for 40 min at 150 V. Each PCR product was isolated using a gel extraction kit (Qiagen), subcloned into a pGEM-T Easy vector (Promega, Madison, WI, USA), and sequenced using a DNA sequence analyzer (ABI 3730, Applied Biosystems Inc., Foster City, CA, USA). The *Sanguisorba* accessions used are listed in Table S7. The primer sequences of S_matK, S_rbcL, MR16, Y1-1, Y1-2, and Y3 are listed in Table S9.

### 4.7. Phylogenetic Analysis

A total of 11 cp genomes, nine from Sanguisorbinae along with *Agrimonia pilosa* and *Hagenia abyssinica* as outgroups, were used for phylogenetic analysis. Of these, eight cp genome sequences were downloaded from the NCBI GenBank database (Table S10). We used two matrices composed of 77 conserved protein-coding genes (CDSs). Using MAFFT ver. 7.388 [118], the CDS sets were aligned. Each aligned gene was extracted using the Geneious software (https://www.geneious.com, accessed on 20 March 2021), and the genes were arranged alphabetically. The concatenated gene dataset was created using the Geneious software and the CDS dataset. The alignment datasets were filtered to remove ambiguously aligned regions using Gblocks ver. 5 [119]. The best-fitting model of nucleotide substitutions was determined using the Akaike information criterion in jModelTest V2.1.10 [120] (Table S11), and the GTR + I + G model was chosen for ML analysis. The GTR + G model was selected for analyzing the BI. The maximum parsimony (MP) analysis was conducted using the PAUP v4.0b10 [121]. The MP searches included 1000 random addition replicates and TBR branch swapping with the MulTrees option. The ML analysis was performed using RaxML v. 8.0.5 [122] with 1000 bootstrap replicates. The BI analysis was carried out using MrBayes 3.2.2 [123], with two independent runs of four simultaneous chains, executed for 5,000,000 generations using the Markov chain Monte Carlo algorithm. Trees were sampled every 5000 generations, and the first 25% were discarded as burn-in. The trees were determined from a 50% majority-rule consensus to estimate the posterior probabilities. The reconstructed trees were visualized using Figtree V.1.4.2 [124].

## 5. Conclusions

This study comprehensively analyzed the floral micromorphological and palynological characteristics and cp genome sequences of the selected species of *Sanguisorba*. Although the floral micromorphology in all examined species of *Sanguisorba* was similar, the outer sepal waxes and characteristics in the hypanthium had diagnostic values. The palynological data were constant. However, only *S. hakusanensis* had tricolporate pollen grains, whereas the other species had hexacolporate pollens. We observed, for the first time, the absence of orbicules in this genus, and this might be a synapomorphic characteristic of the genus *Sanguisorba*.

Furthermore, genetic variant hotspots were used to develop new DNA barcodes for efficient and rapid species identification. The cp of *Sanguisorba* is well conserved. However, we identified the varied genetic diversity and selective pressure for *psbK* in six *Sanguisorba* cp genomes. We successfully established the phylogenetic relationships among *Sanguisorba* using the whole cp genome sequences and coding sequences. We successfully conducted a comprehensive study of the morphological, micromorphological, pollenological, and genomic characteristics and marker development of the four hard-to-distinguish species of *Sanguisorba*. This study provides useful information on *Sanguisorba* species and a basis for further in-depth research into this plant of medicinal value.

## Figures and Tables

**Table 1:** The floral micromorphological and palynological characteristics of the selected species from *Sanguisorba*.

Species	*S. hakusanensis*	*S. officinalis*	*S. stipulata*	*S. tenuifolia*
Floral characteristics				
Outer sepal cell surface	Smooth	Smooth	Smooth	Smooth
Outer sepal waxes	Platelets	Absent	Absent	Absent
Inner sepal cell surface	Striation	Striation	Striation	Striation
Filament length	6–10 mm	1–3 mm	5–7 mm	6–8 mm
Filament shape	Compressed-dilated in the upper part	Filiform	Compressed-dilated in the upper part	Compressed-dilated in the upper part
Hypanthium shape	Ellipsoid	Ellipsoid	Globose	Globose
Disk-like structure *	Moderate	Thickened	Moderate	Reduced
Hypanthium cell surface	Smooth	Smooth	Smooth	Smooth
Hypanthium trichomes	Basal and ventral suture	Upper part	Absent	Upper part
Style length	2–3 mm	0.5–1 mm	1–2 mm	1–2 mm
Style surface	Striation	Striation	Striation	Striation
Stigma shape	Fimbriate with blunted apex	Fimbriate with blunted apex	Fimbriate with acute apex	Fimbriate with acute apex
Stigma surface	Striation	Striation	Striation	Striation
Palynological characteristics				
Aperture shape	Tricolporate	Hexacolporate	Hexacolporate	Hexacolporate
Polar axis (P)	23.6 ± 1.5	16.3 ± 0.8	21.6 ± 1.2	25.0 ± 1.3
Equatorial diameter (E)	21.0 ± 1.3	15.5 ± 1.1	19.6 ± 0.9	23.5 ± 1.6
P/E ratio	1.1 ± 0.1	1.1 ± 0.1	1.1 ± 0.1	1.1 ± 0.1
Shape	Prolate-spheroidal	Prolate-spheroidal	Prolate-spheroidal	Prolate-spheroidal
Colpus width (C)	6.7 ± 1.0	5.8 ± 0.7	7.3 ± 0.9	8.7 ± 0.9
Mesocolpus width (M)	11.3 ± 1.3	6.0 ± 0.6	7.1 ± 0.9	9.4 ± 1.2
C/M ratio	0.6 ± 0.1	1.0 ± 0.2	1.0 ± 0.2	0.9 ± 0.1
Exine ornamentation	Microechinate	Microechinate	Microechinate	Microechinate
Orbicule	Absent	Absent	Absent	Absent

* Disk-like structure located on top of the hypanthium.

**Table 2:** Features of the *Sanguisorba* chloroplast genomes.

Species	*S. officinalis*	*S. stipulata*	*S. hakusanensis*	*S. tenuifolia*	*S. sitchensis*	*S. filiformis*
Accession number	In this study	In this study	In this study	MH513641	NC_044691	NC_044693
Total cp genome size (bp)	155,412	155,403	155,645	155,403	155,127	154,282
Large single-copy (LSC) region (bp)	85,478	85,444	85,697	85,525	85,347	84,405
Inverted repeat (IR) region (bp)	25,573	25,562	25,593	25,576	25,615	25,609
Small single-copy (SSC) region (bp)	18,788	18,760	18,762	18,726	18,550	18,659
Total number of genes (unique)	112	112	112	112	112	112
Protein-coding gene (unique)	78	78	78	78	78	78
rRNA (unique)	4	4	4	4	4	4
tRNA (unique)	30	30	30	30	30	30
GC content (%)	37.2	37.2	37.2	37.2	37.2	37.3
LSC (%)	35.2	35.2	35.2	35.2	35.2	35.3
IR (%)	42.7	42.7	42.7	42.7	42.7	42.8
SSC (%)	31.2	31.2	31.3	31.3	31.4	31.4

**Table 3:** The analysis of DNA barcodes of four *Sanguisorba* species and one variety.

Species	Region	Marke Name	Aligned Length (bp)	Parsimony Informative Site		Nucleotides Diversity (Pi)	Length of Indel	Number of Haplotype
				Number	%			
*Sanguisorba* *	matK	S_matK	689	6	0.9%	0.00296	0	3
	rbcL	S_rbcL	870	7	0.8%	0.00200	0	3
	matK-rps16	MR16	409	5	1.2%	0.00466	22	2
	ycf1	Y1-1	214	5	2.3%	0.00699	0	2
	ycf1	Y1-2	346	0	0.0%	0	12	1
	ycf3 intron	Y3	346	0	0.0%	0	42	1

* *S. hakusanensis*, *S. officinalis*, *S. officinalis* var. *longifolia*, *S. stipulata*, *S. tenuifolia*.

## Data Availability

The data supporting the findings of this study are available within the article and Table 1.

## Supplementary Materials

The following are available online at https://www.mdpi.com/article/10.3390/genes12111764/s1, Figure S1: Distribution of paired-end reads mapped onto complete chloroplast genomes of three *Sanguisorba* species. Figure S2: Circular gene map of chloroplast genomes from *Sanguisorba*. Figure S3: Codon frequencies and RSCU values for six *Sanguisorba* and one *Bencomia* chloroplast genomes. Figure S4: Codon distributions of protein-coding genes in *Sanguisorba* and *Bencomia* chloroplast genomes. Figure S5: Comparison of complete cp genomes of six *Sanguisorba* and one *Bencomia* using the MAUVE algorithm. Figure S6: Comparison of the LSC, IR, and SSC junction positions among *Sanguisorba* and *Bencomia* chloroplast genomes. Figure S7: Ka and Ks values for *Sanguisorba* chloroplast genomes. Figure S8: Ka/Ks values for ATP synthase and Ribosomal proteins related genes in *Sanguisorba* chloroplast genomes. Figure S9: Schematic representation of indel markers for *Sanguisorba* species. Figure S10: ITS DNA barcode sequences alignment images for *Sanguisorba* species. Figure S11: New DNA barcode sequences alignment images for *Sanguisorba* species. Figure S12: Phylogenetic tree based on 77 CDS from six *Sanguisorba* using Bayesian posterior probabilities (PP) and maximum likelihood bootstraps (ML). Table S1: Raw and trimmed read data. Table S2: Genome assembly information for three *Sanguisorba* chloroplast genomes. Table S3: PCR-based sequence validation of chloroplast junctions. Table S4: Genes in the chloroplast genomes of *Sanguisorba* species. Table S5: Genic introns in *S. officinalis*, *S. stipulata*, *S. hakusanensis* chloroplast genomes. Table S6: Genic introns in *S. tenuifolia*, *S. sitchensis*, *S. filiformis* chloroplast genomes. Table S7: Voucher specimen information for floral micromorphology, palynology, and chloroplast genomes, DNA barcode analysis used in this study. Table S8: Primers used in this study for chloroplast junction validation. Table S9: Primer information for DNA barcodes. Table S10: Chloroplast genomes from NCBI used for phylogenetic analysis. Table S11: Best-fitting substitution models selection using jModelTest in CDS sets.
