# Supplementary material for: A Comprehensive Study of the Genus Sanguisorba (Rosaceae) Based on the Floral Micromorphology, Palynology, and Plastome Analysis
Source: Genes (Basel). 2021 Nov 5;12(11):1764. doi: 10.3390/genes12111764 (PMC8618895; doi:10.3390/genes12111764)
Supplement: Supplementary file 1 [file genes-12-01764-s001.zip › genes-1357253-supplementary-done.pdf]

genes

Supplementary Materials: A comprehensive studies of genus *Sanguisorba* (Rosaceae) based on floral micromorphology, palynology, plastome analysis

Inkyu Park†, Jun-Ho Song†, Sungyu Yang, Goya Choi, Byeong Cheol Moon*

Herbal Medicine Resources Research Center, Korea Institute of Oriental Medicine, Naju, 58245, Korea; pik6885@kiom.re.kr (I.P.); songjh@kiom.re.kr (J.-H.S.); sgyang81@kiom.re.kr (S.Y.); serparas@kiom.re.kr (G.C.); bcmoon@kiom.re.kr (B.C.M.)

*Correspondence: bcmoon@kiom.re.kr; Tel.: +82-61-338-7100

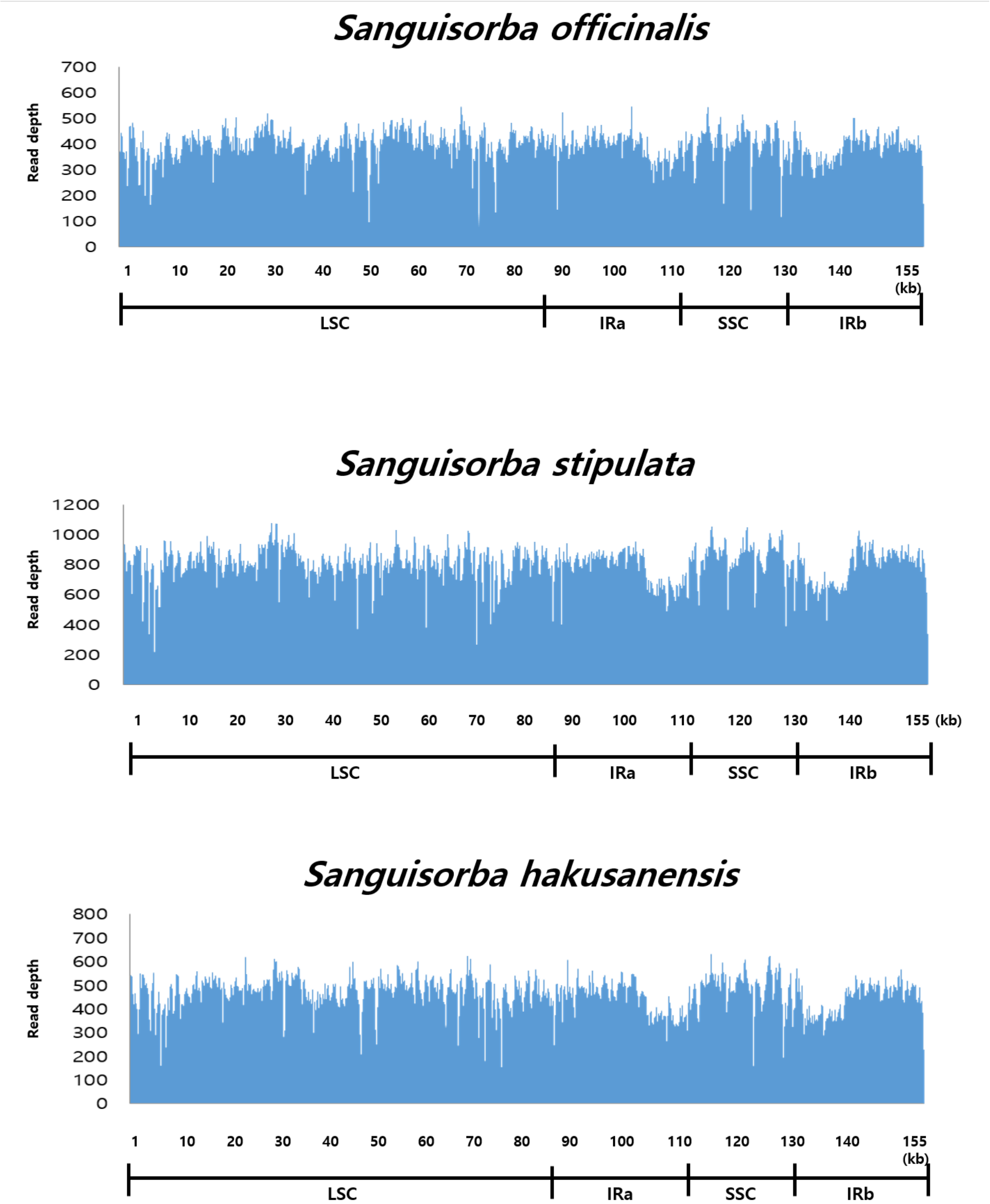

Figure S1: Distribution of paired-end reads mapped onto complete chloroplast genomes of three *Sanguisorba* species. LSC, large single copy region; SSC, small single copy region; IRa, inverted repeat a; IRb, inverted repeat b.

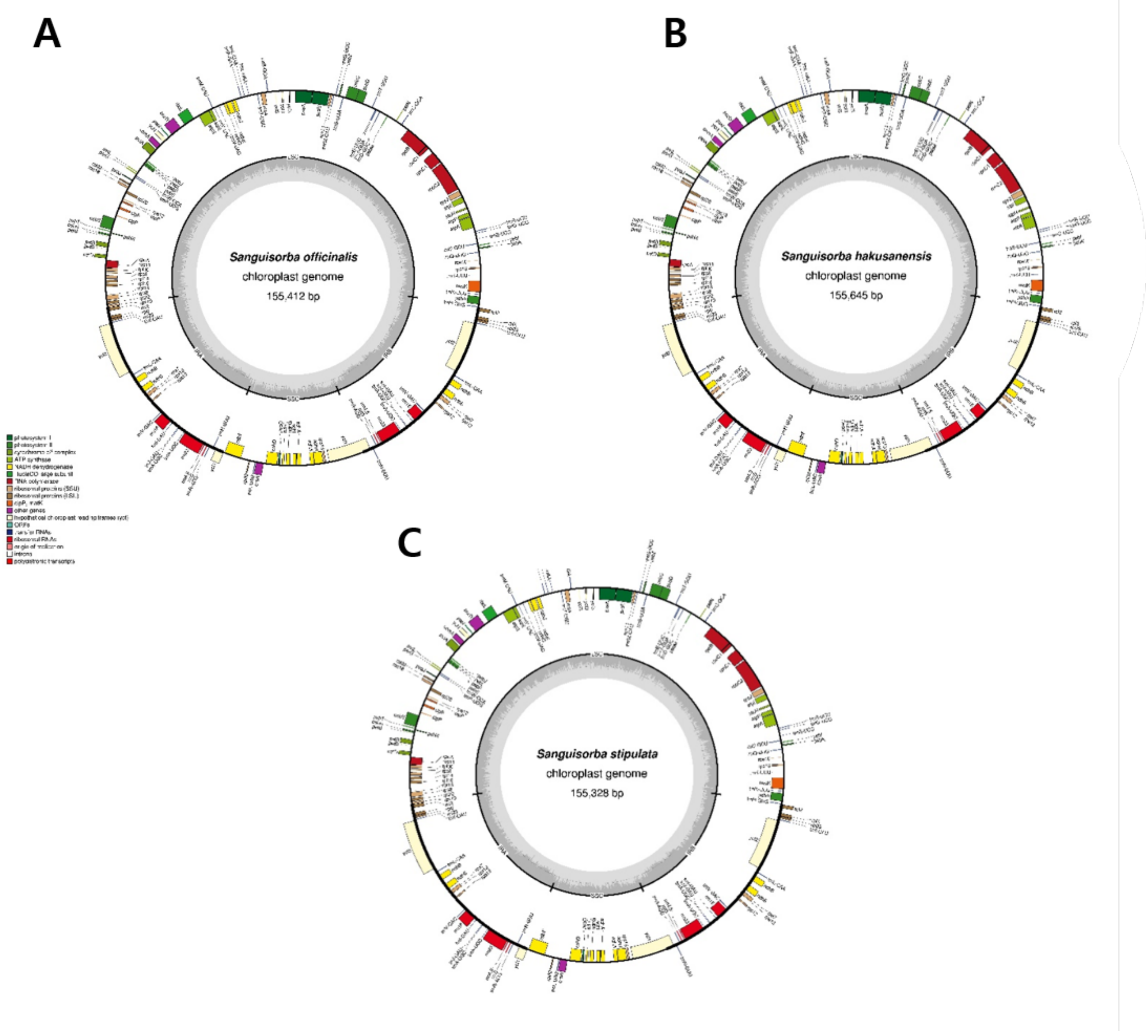

Figure S2: Circular gene map of chloroplast genomes from *Sanguisorba*. (A) *S. officinalis* (B) *S. hakusanensis* (C) *S. stipulata*

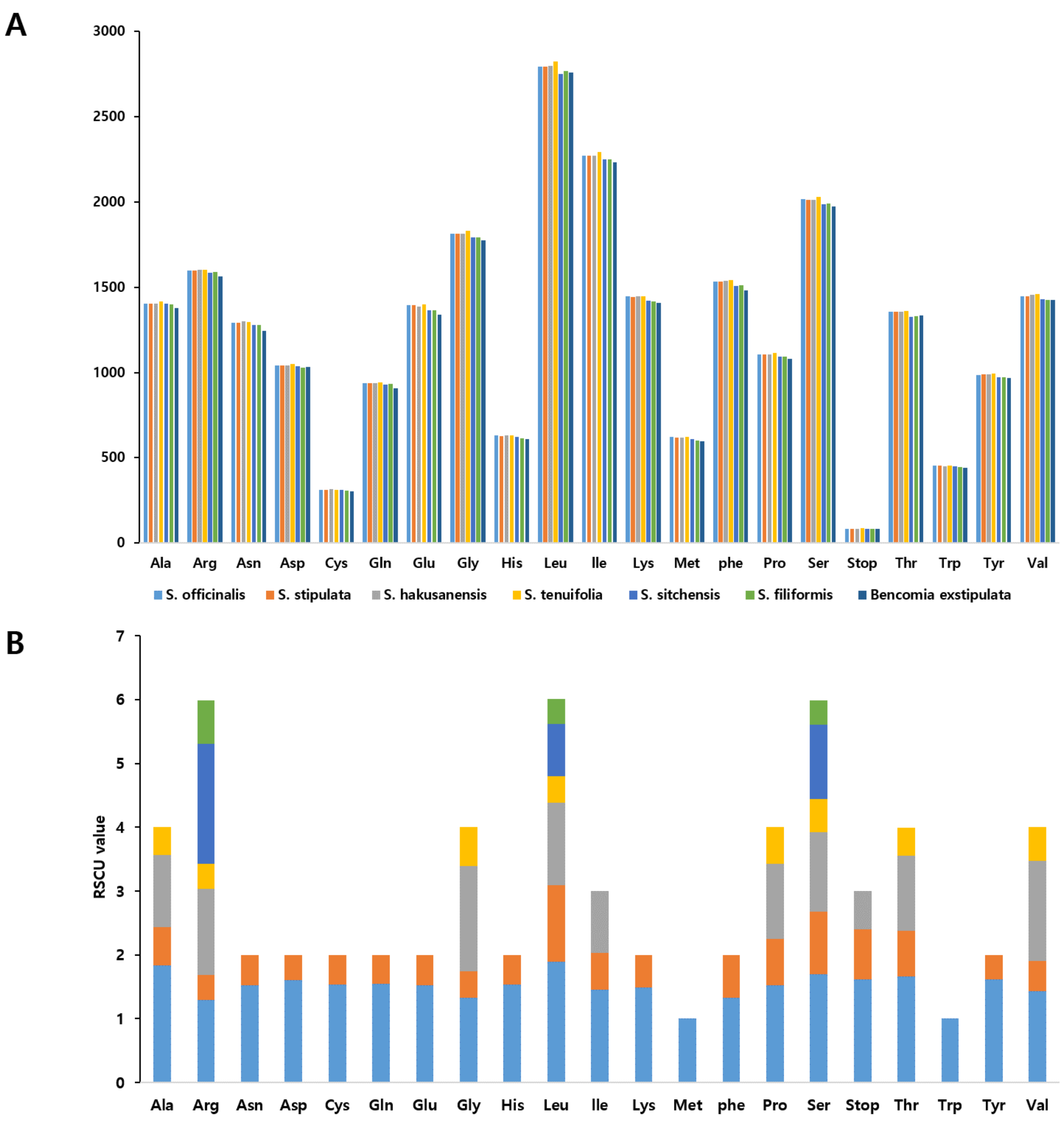

Figure S3: Codon frequencies and RSCU values for six *Sanguisorba* and one *Bencomia* chloroplast genomes. (A) Amino acid frequencies for protein-coding sequences. (B) Codon usage for 20 amino acids and stop codons in 78 protein-coding genes.

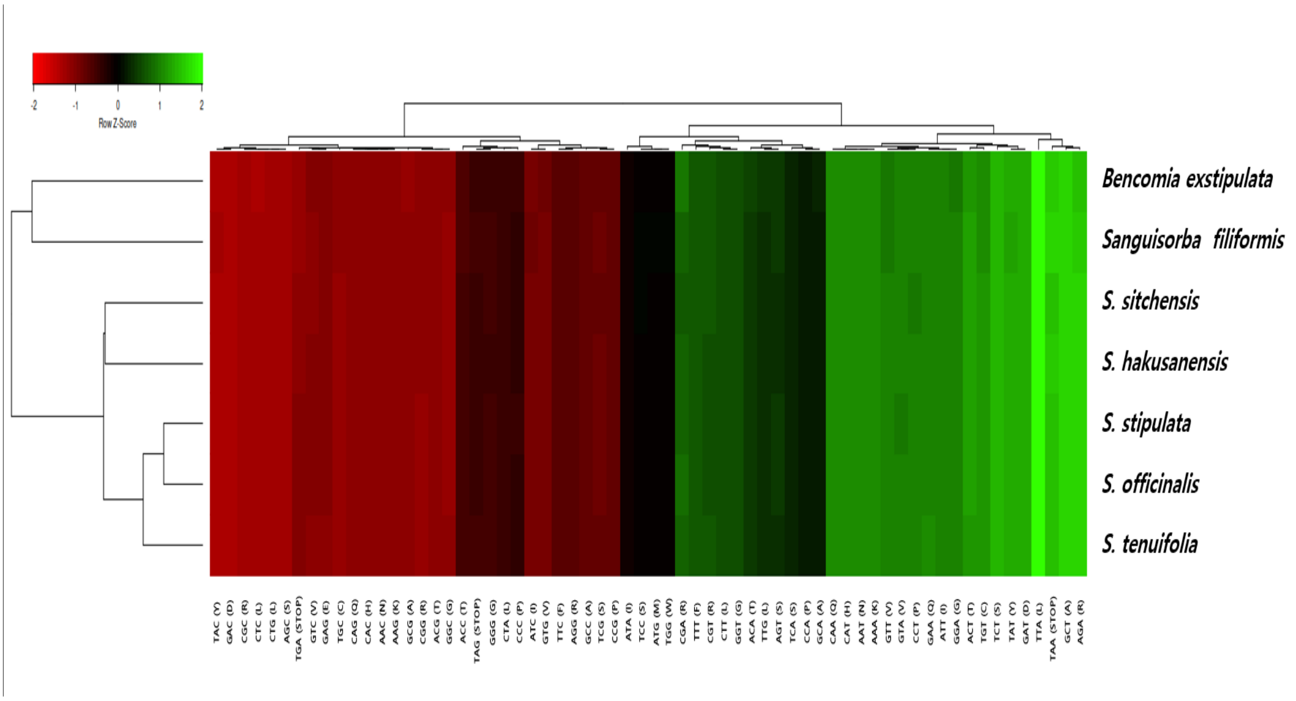

Figure S4: Codon distributions of protein-coding genes in *Sanguisorba* and *Bencomia* chloroplast genomes. Green indicates a high relative synonymous codon usage (RSCU) value and red indicates a low RSCU value. Hierarchical clustering (average linkage method) was performed based on the codon patterns (x-axis).

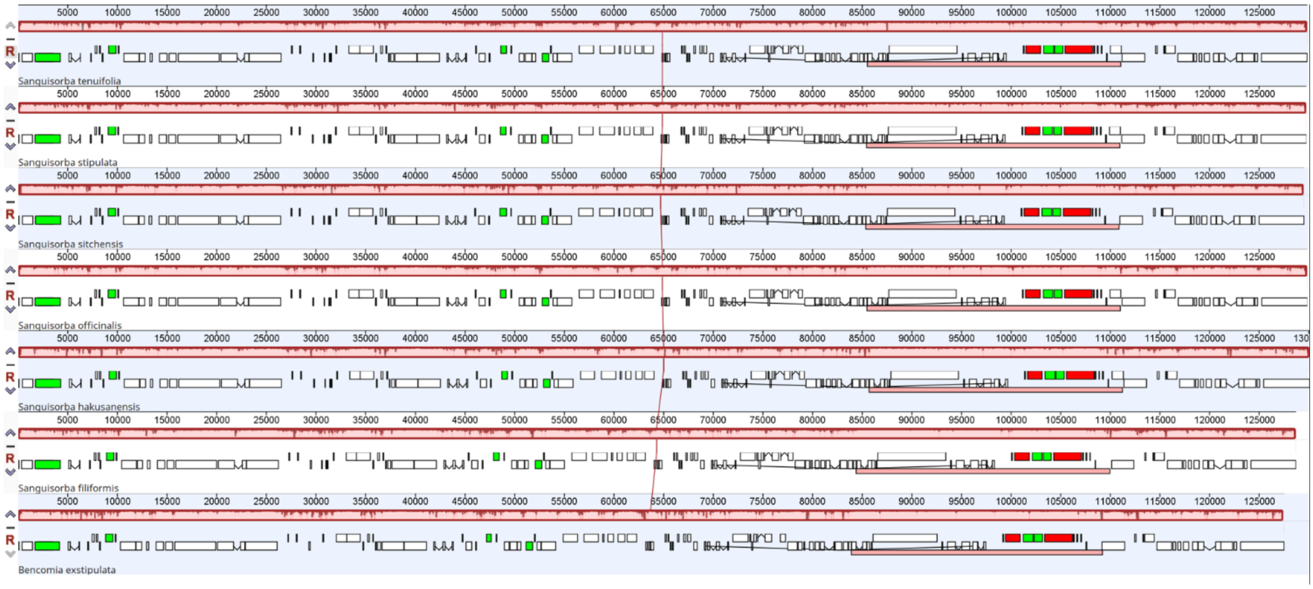

Figure S5: Comparison of complete cp genomes of six *Sanguisorba* and one *Bencomia* using the MAUVE algorithm

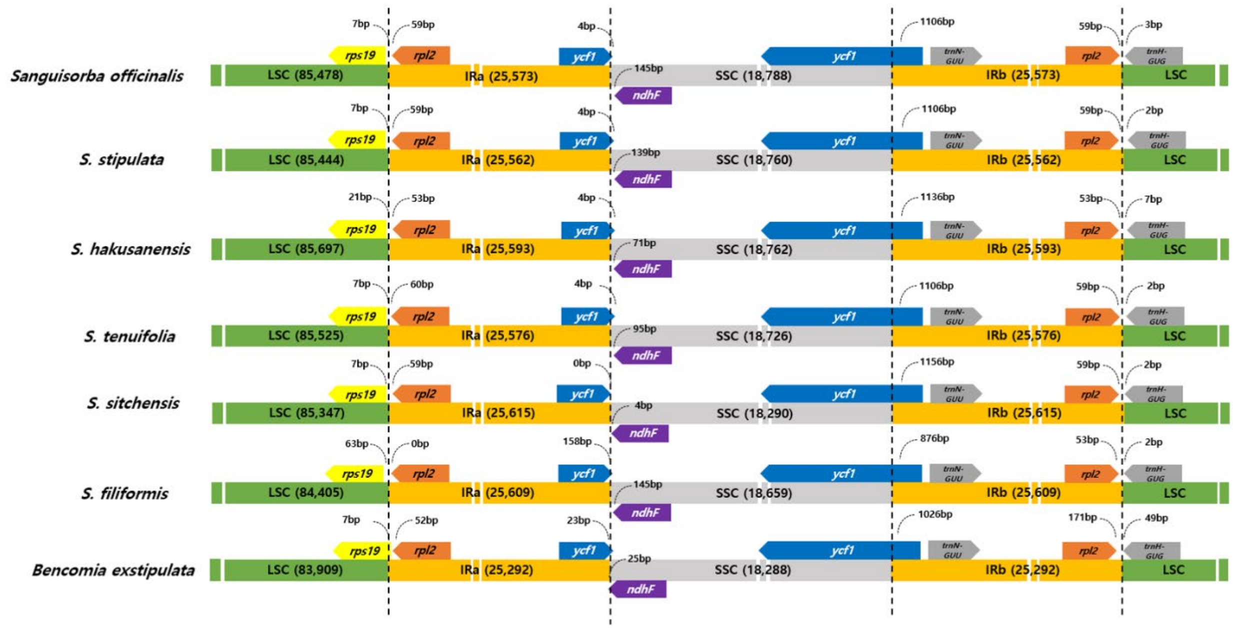

Figure S6: Comparison of the LSC, IR, and SSC junction positions among *Sanguisorba* and *Bencomia* chloroplast genomes.

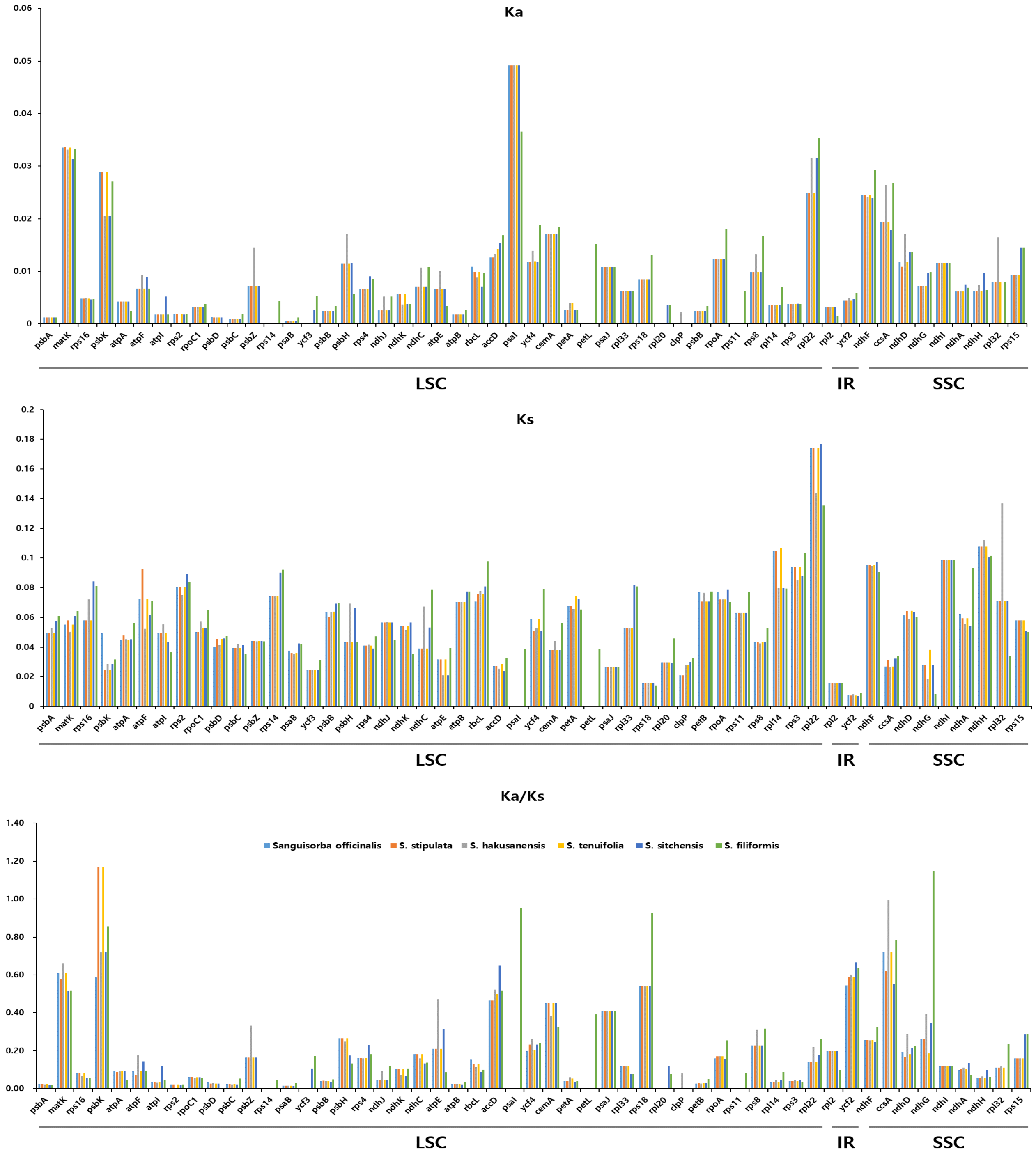

Figure S7: Ka and Ks values for *Sanguisorba* chloroplast genomes. The ratios of non-synonymous substitution (Ka) to synonymous substitution (Ks) were calculated for 78 conserved protein-coding sequences using *Bencomia exstipulata* as a reference. Genes with Ka/ Ks = 0 are not shown.

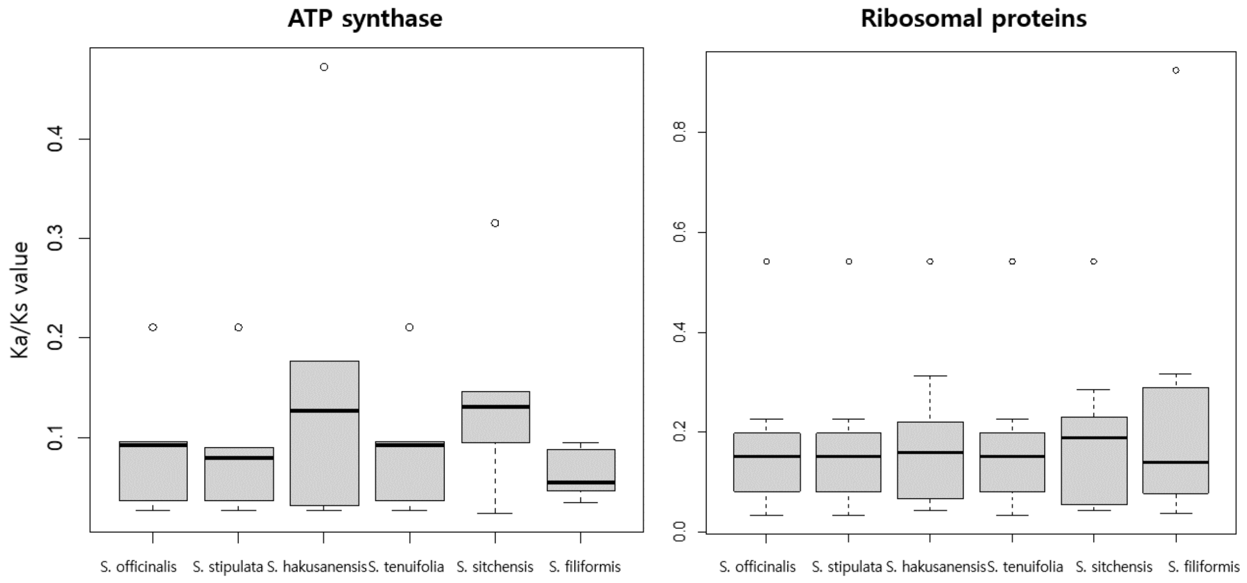

Figure S8: Ka/Ks values for ATP synthase and Ribosomal proteins related genes in *Sanguisorba* chloroplast genomes.

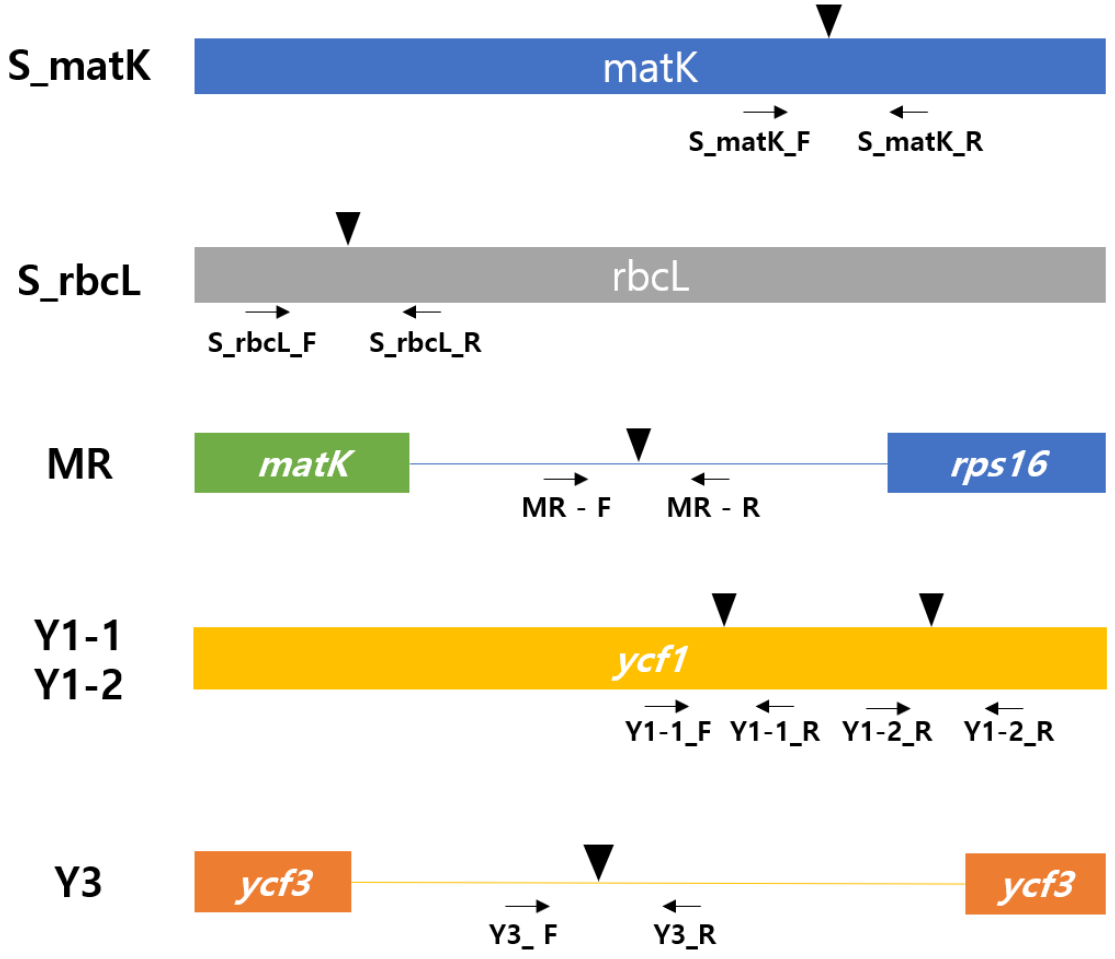

Figure S9: Schematic representation of indel markers for *Sanguisorba* species.

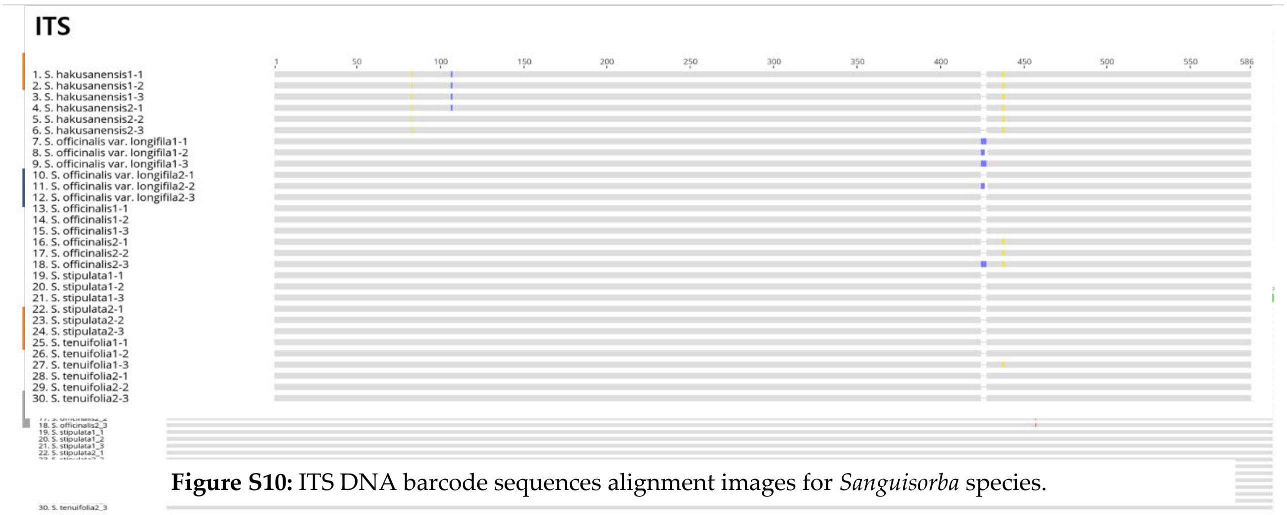

Figure S10: ITS DNA barcode sequences alignment images for *Sanguisorba* species.

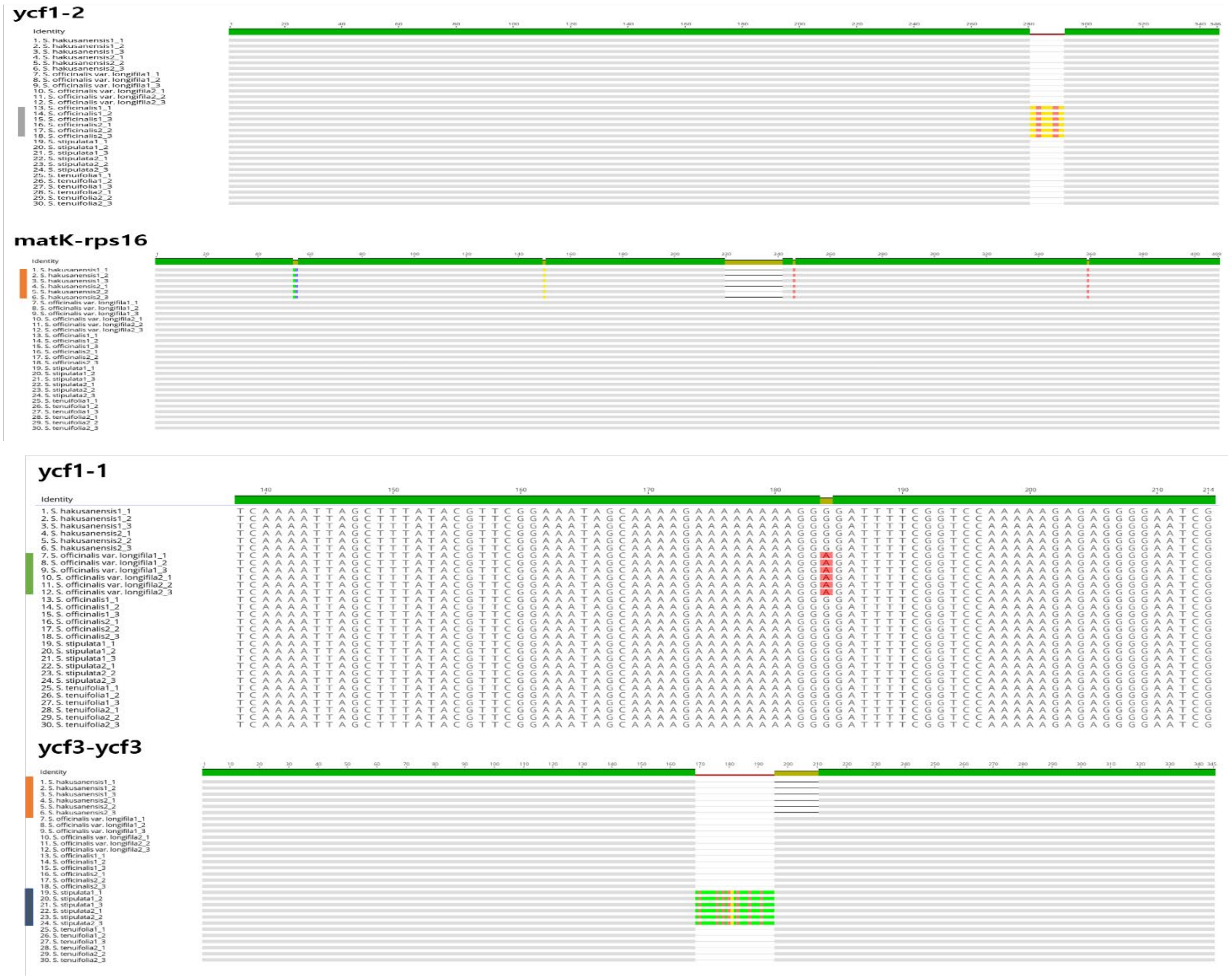

Figure S11: New DNA barcode sequences alignment images for *Sanguisorba* species. Color bar were indicated distinguishing species-specific nucleotides.

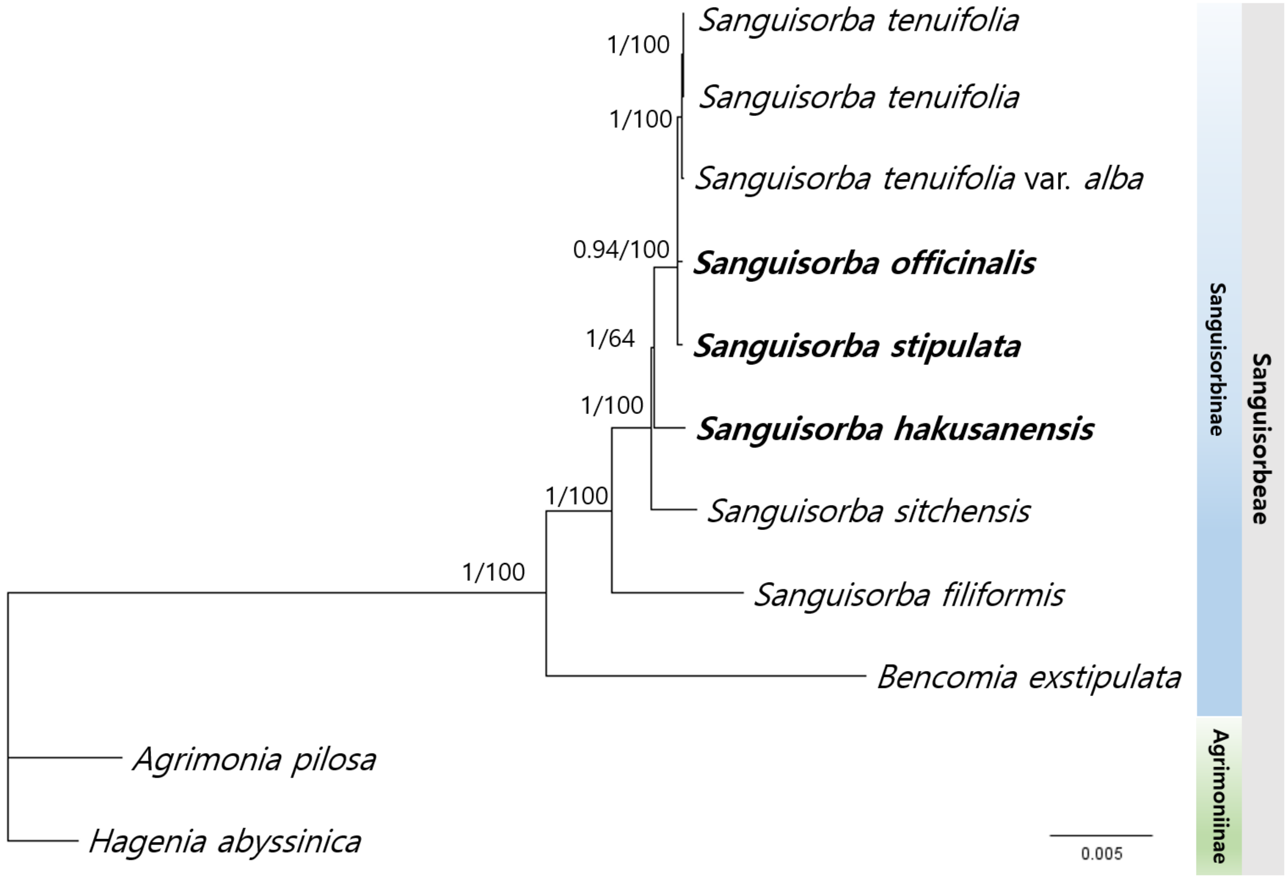

Figure S12: Phylogenetic tree based on 77 CDS from six *Sanguisorba* using Bayesian posterior probabilities (PP) and maximum likelihood bootstraps (ML). PP topology is shown with Bayesian posterior probabilities/ ML bootstrap support values given at each node.

Table S1: Raw and trimmed read data

Scientific name	Input reads	Trimmed reads		Total raw bases	Trimmed bases	
S. officinalis	5,410,282	4,272,854	78.98%	1,573,191,990	975,321,108	62.00%
S. stipulata	8,796,360	7,285,311	82.82%	2,607,611,175	1,694,161,225	64.97%
S. hakusanensis	8,976,704	7,441,653	82.90%	2,667,689,349	1,719,907,973	64.47%

Table S2: Genome assembly information for three *Sanguisorba* chloroplast genomes

Scientific name	Aligned reads (#)	Coverage (x)	Cp genome length (bp)
S. officinalis	258,212	380.66	155,412
S. stipulata	538,692	805.96	155,328
S. hakusanensis	267,460	397.55	155,645

Table S3: PCR-based sequence validation of chloroplast junctions

Species	location	PCR-based sequence (bp)	start	end	Identities	%
S. officinalis	LSC_IRa	436	85,359	85,796	100	
	IRa_SSC	661	110,759	111,419	100	
	SSC_IRb	346	129,799	130,144	100	
	IRb_LSC	590	154,846	23	100	
S. stipulata	LSC_IRa	437	85,325	85,761	100	
	IRa_SSC	678	110,714	111,391	100	
	SSC_IRb	334	129726	130,071	100	
	IRb_LSC	590	154,762	23	100	
S. hakusanensis	LSC_IRa	437	85,406	85,842	100	
	IRa_SSC	610	110,998	111,607	100	
	SSC_IRb	334	130,012	130,357	100	
	IRb_LSC	589	155,085	28	100	

Table S4: Genes in the chloroplast genomes of *Sanguisorba* species

Group of genes	Name of genes
Photosystem I	*psaA, B, C, I, J, ycf3*[2], *ycf4*
Photosystem II	*psbA, B, C, D, E, F, H, I, J, K, L, M, N, T, Z*
Cytochrome b6/f	*petA, B*[1], *D*[1], *G, L, N*
ATP synthase	*atpA, B, E, F*[1], *H, I*
Rubisco	*rbcL*
NADH oxidoreductase	*ndhA*[1], *B*[1,3], *C, D, E, F, G, H, I, J, K*
Large subunit ribosomal proteins	*rpl2*[1,3], *14, 16*[1], *20, 22, 23*[3], *32, 33, 36*
Small subunit ribosomal proteins	*rps2, 3, 4, 7*[3], *8, 11, 12*[2,3,4], *14, 15, 16*[1], *18, 19*
RNA polymerase	*rpoA, B, C1*[1], *C2*
Unknown function protein coding gene	*ycf1*[3], *2*[3]
Other genes	*accD, ccsA, cemA, clpP*[2], *matK*
Ribosomal RNAs	*rrn16*[3], *23*[3], *4.5*[3], *5*[3]
Transfer RNAs	*trnA-UGC*[1,3], *trnC-GCA, trnD-GUC, trnE-UUC, trnF-GAA, trnfM-CAU, trnG-GCC, trnG-UCC*[1], *trnH-GUG, trnI-CAU*[3], *trnI-GAU*[1,3], *trnK-UUU*[1], *trnL-CAA*[3], *trnL-UAA*[1], *trnL-UAG, trnM-CAU, trnN-GUU*[3], *trnP-UGG, trnQ-UUG, trnR-ACG*[3], *trnR-UCU, trnS-GCU, trnS-GGA, trnS-UGA, trnT-GGU, trnT-UGU, trnV-GAC*[3], *trnV-UAC*[1], *trnW-CCA, trnY-GUA*

1) Gene containing a single intron, 2) gene containing two introns, 3) two gene copies in IRs, 4) trans-splicing gene

Table S5: Genic introns in *S. officinalis*, *S. stipulata*, *S. hakusanensis* chloroplast genomes

	Gene	Region	exon I	intron I	exon II	intron II	exon III
1	trnk-UUU	LSC	37	2513, 2516,2511	35		
2	rps16	LSC	39	899, 899, 909	228		
3	trnG-UCC	LSC	23	697	48		
4	rpoC1	LSC	435	749	1620		
5	ycf3	LSC	126	720,720,740	228	766,766,772	153
6	trnL-UAA	LSC	37	554	50		
7	trnV-UAC	LSC	39	601	37		
8	rps12	LSC	114		232		26
9	clpP	LSC	69	917,917,936	291	658,658,656	228
10	petB	LSC	6	839,839,847	579		
11	petD	LSC	8	750,750,728	475		
12	rpl16	LSC	9	1009,1009,1030	402		
13	rpl2	LSC	393	670	435		
14	ndhB	IR	777	682	756		
15	trnI-GAU	IR	42	949,949,950	35		
16	trnA-UGC	IR	38	814	35		
17	ndhA	SSC	552	1194,1194,1237	540		

Table S6: Genic introns in *S. tenuifolia*, *S. sitchensis*, *S. filiformis* chloroplast genomes

	Gene	region	exon I	intron I	exon II	intron II	exon III
1	trnk-UUU	LSC	37	2511, 2508, 2508	35		
2	rps16	LSC	38	902, 904, 902	229		
3	trnG-UCC	LSC	23	698, 697, 698	48		
4	rpoC1	LSC	435	750,749,758	1620		
5	ycf3	LSC	126	723,729,719	228	766,767,772	153
6	trnL-UAA	LSC	37	554,574,554	50		
7	trnV-UAC	LSC	39	601	37		
8	rps12	LSC	114		232		26
9	clpP	LSC	69	943,821,876	291	660,656,657	228
10	petB	LSC	6	839,760,753	579		
11	petD	LSC	8	750,727,725	475		
12	rpl16	LSC	9	1011,1006,953	402		
13	rpl2	LSC	393	670,673,673	435		
14	ndhB	IR	777	676,682,676	756		
15	trnI-GAU	IR	42	949,950,950	35		
16	trnA-UGC	IR	38	814,812,811	35		
17	ndhA	SSC	552	1197,1179,1192	540		

Table S7: Voucher specimen information for floral micromorphology, palynology, and chloroplast genomes, DNA barcode analysis used in this study

No.	Species	Collection information	Coordinates	Collector *collection no.* (Herbarium acronym)
1		Deogyusan Mt., Seolcheon-myeon, Muju-gun, Jeollabuk-do, Korea	35°51'27.9"N 127°43'18.9"E	B. Choo & Y. Ji *KIOM200901002023* (KIOM)$^{C, M, P, D}$
2	*S. hakusanensis*	Cheonhwangsan Mt., Icheon-ri, Sangbuk-myeon, Ulju-gun, Ulsan, Korea	35°33'29.4"N 128°58'26.4"E	S.C. Ko *KIOM201601018272* (KIOM)$^{M, P, D}$
3		Baegunsan Mt., Gwangyang-si, Jeollanam-do, Korea	35°03'06.1"N 127°35'31.8"E	Y. Ji & B. Moon *KIOM201101004181* (KIOM)$^{M, P}$
4	*S. officinalis*	Nomoksan Mt., Sabuk-ri, Sabuk-eup, Jeongseon-gun, Gangwon-do	37°14'36.2"N 128°51'22.6"E	S. Yang & B. Moon *MBC_KIOM-2016-271* (KIOM)$^{C, M, P, D}$

5		Hwanggeumsan Mt., Sangju-si, Gyeongsangbuk-do, Korea	36°27'46.7"N 128°16'37.6"E	S. Yang & B. Moon *KIOM201301006325* (KIOM)[M, P, D]
6		Handaeoreum Mt., Aewol-eup, Jeju-si, Jeju-do, Korea	33°21'48.1"N 126°25'29.0"E	Y. Ji & B. Moon *KIOM201101003975* (KIOM)[M, P]
7	*S. officinalis* var. *longifolia*	Laobaishan Mt., Huangnihe, Dunhua, Yanbian, Jilin, China	44°31'38.0"N 128°10'38.3"E	B. Moon & W. Jin *KIOM201501013873* (KIOM)[D]
8		Laobaishan Mt., Huangnihe, Dunhua, Yanbian, Jilin, China	44°31'38.0"N 128°10'38.3"E	B. Moon & W. Jin *KIOM201501013874* (KIOM)[D]
9	*S. stipulata*	Changbaishan Mt., Jilin, China	41°57'54.7"N 127°47'12.2"E	B. Choo & Y. Ji *KIOM200901001889* (KIOM)[C, M, P, D]
10		Changbaishan Mt., Jilin, China	41°57'54.7"N 127°47'12.2"E	B. Choo & Y. Ji *KIOM200901001890* (KIOM)[M, P, D]
11		Changbaishan Mt., Jilin, China	41°57'54.7"N 127°47'12.2"E	B. Choo & Y. Ji *KIOM200901001891* (KIOM)[M, P]
12	*S. tenuifolia*	Seoraksan Mt., Buk-myeon, Inje-gun, Gangwon-do	38°07'58.0"N 128°15'34.8"E	S. Yang & B. Moon *MBC_KIOM-2016-143* (KIOM)[C, M, P, D]
13		Haenam-eup, Haenam-gun, Jeollanam-do, Korea	34°33'56.0"N 126°35'09.9"E	S. Yang & B. Moon *KIOM201201005141* (KIOM)[M, P, D]

[C], chloroplast genomic study; [M], micromorphology; [P], palynology; [D], DNA barcode analysis

Table S8: Primers used in this study for chloroplast junction validation

Primer name	Primer sequence (5`>3`)	Position
LI_F	GACGCCCGAGACCAAGTTAT	LSC_IRa
LI_R	GTCGGACAAGTGGGGAATGT	
IS_F	ACTTCCGAAACGAAGGGGAC	IRa_SSC
IS_R	AATCGCGGTTACATAGATGTTTTT	
SI_F	GTAAGGGCCGATTCCATCGT	SSC_IRb
SI_R	ACTTCCGAAACGAAGGGGAC	
IL_F	GCCGTATGCTTTGGAAGAAGC	IRb_LSC
IL_R	GTTCAATTCCCGTCGTTCGC	

Table S9: Primer information for DNA barcodes

Primer name	Primer sequence (5`>3`)	Position	annealing temperature (°C)
S_matK_F	ACCCTTCGCTACTCCGTGAA	matK	60
S_matK_R	TCCGCCCAGCTGGACTTACT		
S_rbcL_F	GGTTCAAGGCCTTACGCGCT	rbcL	61
S_rbcL_R	CAGGTGCATTTCCCCAAGGGT		
MR_F	GACCCATTTATCCACGATCGGA	matK-rps16	59
MR_R	TGACGTATTGAAGTAATGGGGTCT		
Y1-1_F	AACACTATCCCAGGCTTCCG	ycf1	59
Y1-1_R	CCCCTCTCTTTTTGGACCGA		
Y1-2_F	GTAAGGGCCGATTCCATCGT	ycf1	59
Y1-2_R	ACTTCCGAAACGAAGGGGAC		
Y3_F	ACGGCGCTTCCTCTATCAAT	ycf3 intron	60
Y3_R	GGCACTAGAACGAAACCCGT		

Table S10: Chloroplast genomes from NCBI used for phylogenetic analysis

No.	Tribe	Sub-tribe	Taxon	GenBank accession number
1	Sanguisorbeae (Family Rosaceae)	Sanguisorbinae	*Sanguisorba tenuifolia*	NC_042223
2			*Sanguisorba tenuifolia*	MH513641
3			*Sanguisorba tenuifolia* var. *alba*	NC_044692
4			*Sanguisorba sitchensis*	NC_044691
5			*Sanguisorba filiformis*	NC_044693
6			*Bencomia exstipulata*	NC_039924
7		Agrimoniinae	*Agrimonia pilosa*	NC_050051
8			*Hagenia abyssinica*	KX008604

Table S11: Best-fitting substitution models selection using jModelTest in CDS sets

	Model	f(a)	f(c)	f(g)	f(t)	kappa	titv	Ra	Rb	Rc	Rd	Re	Rf	pInv	gamma
AIC	GTR+I+G	0.3	0.1	0.1	0.3	0	0	0.9	1.4	0.2	0.5	1.4	1	0.47	0.65

Model	-lnL*	K	AIC	Delta	weight	cumWeight
GTR+I+G	258526.7	31	517115.4	0	0.664254	0.664254
TVM+I+G	258526.4	32	517116.8	1.3965	0.330437	0.994691
GTR+G	258533.2	30	517126.3	10.89614	0.002859	0.99755
TVM+G	258533	31	517128	12.57516	0.001235	0.998785
GTR+I	258534.4	30	517128.7	13.32534	0.000849	0.999634
TVM+I	258534.2	31	517130.4	15.00768	0.000366	1
TPM1uf+I+G	258597.8	29	517253.6	138.1589	6.63E-31	1
TIM1+I+G	258597.7	30	517255.3	139.9078	2.77E-31	1
TIM3+I+G	258604.1	28	517264.1	148.7269	3.36E-33	1
TPM3uf+I+G	258604.9	28	517265.7	150.3199	1.52E-33	1
